# GWAS and RNA-seq analysis uncover candidate genes associated with alkaline stress tolerance in maize (*Zea mays* L.) seedlings

**DOI:** 10.3389/fpls.2022.963874

**Published:** 2022-07-18

**Authors:** Chunxiang Li, Yue Jia, Runyu Zhou, Liwei Liu, Mengna Cao, Yu Zhou, Zhenhua Wang, Hong Di

**Affiliations:** Key Laboratory of Germplasm Enhancement, Physiology and Ecology of Food Crops in Cold Region, Ministry of Education, Northeast Agricultural University, Harbin, China

**Keywords:** maize, alkali tolerance, genome-wide association study, RNA-seq, candidate genes

## Abstract

Soil salt-alkalization is a common yet critical environmental stress factor for plant growth and development. Discovering and exploiting genes associated with alkaline tolerance in maize (*Zea mays* L.) is helpful for improving alkaline resistance. Here, an association panel consisting of 200 maize lines was used to identify the genetic loci responsible for alkaline tolerance-related traits in maize seedlings. A total of nine single-nucleotide polymorphisms (SNPs) and their associated candidate genes were found to be significantly associated with alkaline tolerance using a genome-wide association study (GWAS). An additional 200 genes were identified when the screen was extended to include a linkage disequilibrium (LD) decay distance of r^2^ ≥ 0.2 from the SNPs. RNA-sequencing (RNA-seq) analysis was then conducted to confirm the linkage between the candidate genes and alkali tolerance. From these data, a total of five differentially expressed genes (DEGs; |log2FC| ≥ 0.585, *p* < 0.05) were verified as the hub genes involved in alkaline tolerance. Subsequently, two candidate genes, *Zm00001d038250* and *Zm00001d001960,* were verified to affect the alkaline tolerance of maize seedlings by qRT-PCR analysis. These genes were putatively involved protein binding and “flavonoid biosynthesis process,” respectively, based on Kyoto Encyclopedia of Genes and Genomes (KEGG) and Gene Ontology (GO) enrichment analyses. Gene promoter region contains elements related to stress and metabolism. The results of this study will help further elucidate the mechanisms of alkaline tolerance in maize, which will provide the groundwork for future breeding projects.

## Introduction

Soil salt-alkalization is a major source of stress to plants, and therefor a threat to agricultural development and crop productivity (Zhu, 2016). Due to the increase in global temperature caused by human activity and industrial development, the serious evaporation of soil water, and the lack of drainage consideration during irrigation has led to the deepening of land salinization, threatening crop yields. The effect of salty soils on plants includes salt stress caused by neutral salt (NaCl and Na_2_SO_4_; Xu et al., 2019), alkaline salt stress caused by alkaline salt (NaHCO_3_ and Na_2_CO_3_; Yang et al., 2019), and saline-alkali stress caused by both alkaline and neutral salt (Shi and Sheng, 2005). Several studies have shown that alkaline salts, due to their high pH, are more dangerous to plants than neutral salts (Campbell and Nishio, 2000; Hartung et al., 2002; Chen et al., 2012).

Saline-alkali stress induces a variety of plant responses, including changes in plants’ physiological, biochemical, and morphological structures. It also causes ionic imbalance and changes in osmotic pressure (100 mM NaCl; Chaparzadeh et al., 2004; Ambede et al., 2012; Abreu et al., 2013; Mishra et al., 2013). For example, stress accumulation of Na^+^ leads to competition with K^+^ for protein binding, which inhibits protein synthesis (Schachtman and Liu, 1999; Pardo and Quintero, 2002). Physiologically speaking, salt stress reduced stomatal number, stomatal density, and photosynthesis, and increased the respiration rate in plants (100 and 200 mM NaCl) (Dikobe et al., 2021). Biochemically, enzyme content such as amount of soluble proteins, total free amino acids, prolines, Na^+^, and malondialdehyde was increased under alkaline treatment (75 mM Na_2_CO_3_) (Abdel Latef and Tran, 2016). Saline soil also leads to decreased seed vigor, reduced germination, damaged root cell structure, reduced nutrient absorption and utilization, and ultimately reduced yield as a culmination of all these factors (mixed salts of NaCl, Na_2_SO_4_, NaHCO_3_, and Na_2_CO_3_; Peng et al., 2008; Gao et al., 2014). For example, under different concentrations of saline irrigation, maize yield decreased by 2.08%–3.93% with an increase of the salt concentration (NaCl; Feng et al., 2017). Soil salinization affected the physiological and biochemical indicators, or other resistance, ultimately leading to yield impacts.

Maize (*Zea mays* L.) is an important cereal crop in the world. Among the cereal crops, it is the most sensitive to alkalinity. Therefore, exploiting genes associated with alkaline tolerance is helpful for improving alkaline resistance in this crop plant. However, not many genes have been associated with alkaline tolerance. Currently, there has been some progress in identifying the genetic loci associated with these tolerances in crop plants. For instance, a major quantitative trait locus (QTL) for photosynthetic alkali tolerance was detected for two consecutive years by the RIL population in rice (8.5 mM Na_2_CO_3_; Sun et al., 2019). Three pairs of additive by additive (AA) epistatic QTLs associated with dead leaf rate under alkali tolerance were also identified (pH 8.7 to 8.9, natural alkaline soil; Liang et al., 2014). In maize, researchers identified two QTLs associated with alkali tolerance by using simple sequence repeats (SSR) and specific locus amplified fragment-sequencing (SLAF-seq) markers through 151 F_2:3_ populations (100 mM Na_2_CO_3_; Zhang et al., 2018). For example, the researchers mapped a major QTL for alkali tolerance on Chr 2 in maize by identifying sodium ion content and validating the candidate gene (100 mM NaHCO_3_; Cao et al., 2020). When maize was treated with alkaline salt, the ZmNSA1 protein bound to Ca^2+^ for degradation and made H^+^ efflux, ultimately promoting saline-alkaline tolerance in the treated plants (100 mM NaHCO_3_ or NaCl; Cao et al., 2020).

In addition to using QTLs to discover alkaline stress-related traits, GWAS have also been used to detect several SNPs associated with this trait. GWAS has been shown to be an effective method to identify genes and alleles associated with certain agronomic traits under complex environments (Yan et al., 2011; Li et al., 2016). Depending on the rapid decay of LD and amount of diversity, GWAS provides a systematic approach to the analysis of complex quantitative traits in many crops, including maize (Li et al., 2013). In the whole wheat genome, 326,570 SNPs were used in the SNP-GWAS method to unearth 20 SNPs that were significantly associated with grain length and 31 SNPs with grain width, among which the IWB32119 marker located on chromosome 2A was associated with the grain weight locus gene *TaFlo2-A1* (Sajjad et al., 2017; Li et al., 2019). As a benefit of the rapid decay of maize LD, numerous loci controlling complex traits have been identified in maize by GWAS. Researchers extracted two candidate genes associated with low-temperature tolerance in maize by GWAS mining and used RNA-seq data analysis to confirm that these genes were in fact related to low-temperature tolerance (Zhang et al., 2020). Seven candidate genes associated with drought were identified by combining GWAS, DEGs, and co-expression analysis (Guo et al., 2020). Development of the functional SNP marker Sh2_rs844805326 for the prediction of sweet corn was achieved using GWAS (Ruanjaichon et al., 2021). GWAS has also been used to mine genes that control complex traits in maize, such as leaf development, plant height, and ear height (Li et al., 2016; Miculan et al., 2021). As useful as the GWAS method is, there are few reports on the mining of maize alkali tolerance-related genes using GWAS.

In this study, a panel of 200 different inbred maize lines was used to analyze ten traits associated with alkali resistance by GWAS. Candidate genes were identified and further confirmed using RNA-seq data find the following: (a) SNPs associated with resistance to alkaline, (b) alkali tolerance maize inbred lines, and (c) relevant candidate genes for future agricultural research and breeding applications.

## Materials and methods

### Plant materials

A total of 200 inbred maize lines with extensive variation in yield traits and biotic stress tolerances were analyzed in this study (Supplementary Table S1; Weng et al., 2011; Liu et al., 2015; Zhang et al., 2017). The seeds were planted in Harbin, Heilongjiang Province, and produced by manual self-pollination. The field site used was well-managed and free from pests and diseases. After seeds were harvested and dried completely, they were stored in a 4°C seed cabinet until ready for use. The 200 maize inbred lines had a >90% germination rate at 25°C, as shown by a previous study conducted. The alkaline-resistant maize line K10 was analyzed using RNA-seq to assess the expression levels of the whole genome.

### Seed germination and alkaline stress treatment during seedling stage

To germinate the maize seeds, they were first sterilized in 10 g/l sodium hypochlorite for 20 min. After rinsing the seeds three times with sterilized distilled water, they were imbibed for 6 h at 25°C, then the seeds were subjected to the standard germination: briefly, 30 sterile seeds were placed between two wet germination papers (Li et al., 2018). Seed germination was defined as the observation of a 0.5 cm radicle emergence. After 48 h of seed germination, the seedlings with the same bud length were selected and transferred to pots to continue to grow, with 5 seedlings per pot and 30 seedlings per treatment. Seeds were grown in the growth chamber with an 18 h, 25°C/6 h, 22°C light/dark, and temperature/light cycle (Ma et al., 2020). When the seedlings reached the three-leaf growth stage, they were treated with ½ Hoagland solution containing 25 mmol/l Na_2_CO_3_ every day for 10 days (Geng et al., 2020). The control plants were also irrigated with ½ Hoagland solution. The treatment group was watered with ½ Hoagland every 30 days to prevent the excessive accumulation of Na_2_CO_3_. Three independent experiments were used for each treatment and the control.

### Phenotypic data measurement and analysis

After Na_2_CO_3_ treatment for 10 days, maize roots were collected, rinsed with deionized water, and dried by careful blotting before measurements were taken. For root trait data analysis, the Epson Perfection V800 scanner was used to measure root traits to be analyzed by the Regent WinRHIZO (Canada) software. The root traits were root length (RL), root average diameter (RAD), root surface area (RSA), root volume (RV), and root tip number (RTN). Root fresh weight (RFW) and shoot fresh weight (SFW) were measured immediately after observing no moisture in the seedlings. After drying at 105°C for 30 min, roots were transferred to 80°C to continue to dry until they reached a constant weight, then the root dry weight (RDW) and shoot dry weight (SDW) were measured. A ruler was used to measure seed length (SL). The relative performance of the 10 traits was used as an indicator of alkali tolerance. Different treatments consisted of 30 seedlings. Three seedlings were measured for each trait, and the average value of these measurements was taken.

Phenotypic data for the mean, maximum, minimum, kurtosis, skewedness, variance analysis, and standard deviation for each relative trait were analyzed by IBM SPSS Statistics (20.0).^1^ Correlation analysis of phenotype data was performed with the “Performance Analytics” package in R and IBM SPSS Statistics (20.0). ANOVA for 10 alkali tolerance-related traits was calculated using IBM SPSS Statistics (20.0). The broad-sense heritability (H^2^) was estimated using the following formula: H^2^ = V_G_/V_G_ + V_E_, with H^2^ as the heritability estimate, V_G_ as the genotype variance, and V_E_ as the environmental variance.

### Genotypic data and GWAS analysis

Using the Illumina BeadStation 500 G SNP developed by Illumina, 41,110 SNPs were selected from 56,110 SNPs with a minor allele frequency (MAF) > 0.05. SNPs with <20% heterozygosity were used for analysis (Weng et al., 2011; Liu et al., 2015; Zhang et al., 2017). From this, a total of 40,697 SNPs were given for association analysis. 7,742 distributed SNP datasets were assessed for structural parameters by using the STRUCTURE 2.3 software (Evanno et al., 2005). ΔK was calculated using Structure Harvester (Earl and vonHoldt, 2011). Using the software TASSEL 5.0, the kinship information for the 200 inbred lines was estimated.

Using the MLM model in TASSEL 5.0 (Bradbury et al., 2007), the association analysis was performed on the 10 relative traits measured at the seedling stage, using 0.05/Ne to calculate the threshold for association analysis. Because the Bonferroni correction (0.05/41,110 = 1.22e-6) was too conservative, 10 trait-associated loci were considered rare; therefore, the use of a less stringent threshold of −log10(P) > 4 was applied for detecting significant associations (Weng et al., 2011; Zhang et al., 2017; Jia et al., 2020). The Manhattan plot was then generated with the CMplot package in R. When r^2^ = 0.1, the LD decay was 55, 60, 110, 100, 60, 45, 80, 70, 60, and 110 across chromosomes 1–10, respectively. At r^2^ = 0.2, the LD decay was 395, 520, 710, 610, 770, 575, 775, 775, 1,125, and 850 across chromosomes 1–10, respectively (Zhang et al., 2020). Significantly associated SNPs with a physical distance less than the LD decay distance that were also located on the same chromosome were defined as one locus. The upstream and downstream ranges of the corresponding LD decay distances for each locus were used to further mining genes. The genetic information of candidate genes was retrieved using the gene model from the B73 RefGen_V4 in the MaizeGDB website.^2^

### Transcriptomic analysis

The maize inbred line K10, which had high alkaline tolerance, was used for RNA-seq analysis. Before germination, the seeds were treated with the same sterilization method described in section Seed Germination and Alkaline Stress Treatment During Seedling Stage. The treated and control groups were sampled after 3 and 4 days Na_2_CO_3_ treatment, at the three-leaf stage. Leaves were taken from each plant, frozen in liquid nitrogen, and cryopreserved at −80°C until RNA extraction with Trizol. The maize RNA was sent to DATA Corporation (Beijing, China) for library construction. Genomic DNA was removed using DNase I and mRNA was enriched using oligo (dT) magnetic beads and subsequently fragmented. The fragmented products were then amplified by PCR and sequenced after the samples passed the in-house quality inspection.

For our data analysis, the maize genome ZmB73RefGenv4^3^ was used as the reference genome. Gene expression was calculated using Cuffdiff software and expressed as fragments per kilobase of transcript per million fragments mapped (FPKM) value. The threshold for the significant differential expression of genes was set to *p* < 0.05 and |log2FC| ≥ 0.585.

### Identification and annotation of candidate genes

GO enrichment analysis was used to identify all GO terms for DEGs that were significantly enriched in background genes. All DEGs utilized publicly available databases^4^ for GO term classification and grouping. A hyper-geometric test and Phytozome^5^ were used to identify significantly enriched GO terms. GO terms were divided into three categories: “biological process,” “cellular component,” and “molecular function” (Du et al., 2010). To further understand the biological functions of DEGs, enrichment analysis was performed using the KEGG pathway^6^ (Kanehisa et al., 2008). Genes were annotated using the MaizeGDB and NCBI^7^ databases. Heatmaps were plotted using an online site.^8^

### Quantitative real-time PCR analysis

A qRT-PCR assay was performed using the same total RNA used for the transcriptome data analysis of the alkaline-tolerant inbred line K10 and the alkaline-sensitive inbred line Mo17. For each sample, the *TransScript*^®^ One-Step gDNA Removal Kit and cDNA Synthesis SuperMix (TransGen Biotech, Beijing, China) was used to synthesize cDNA. The real-time PCR System (Analytik Jena, Germany) was used for qRT-PCR analysis. As a control, actin was used to normalize the obtained Ct values. Primer5.0 was used to design specific primers for this assay (Supplementary Table S2). The 20 μl reaction mixtures consisted of 2 μl cDNA, 7.2 μl of ddH2O, 0.4 μl each of the forward and reverse primers (10 μM), and 10 μl of 2 × *TransStart*^®^ Tip Green qPCR SuperMix (TransGen Biotech, Beijing, China). Relative gene expression was calculated using the 2^−ΔΔCT^ analytical method (Livak and Schmittgen, 2001). Three biological replicates were combined to be used for three technical replicates, and the average of the three replicates was used for gene expression analysis.

## Results

### Phenotypic traits related to alkaline tolerance

**Figure 1:**
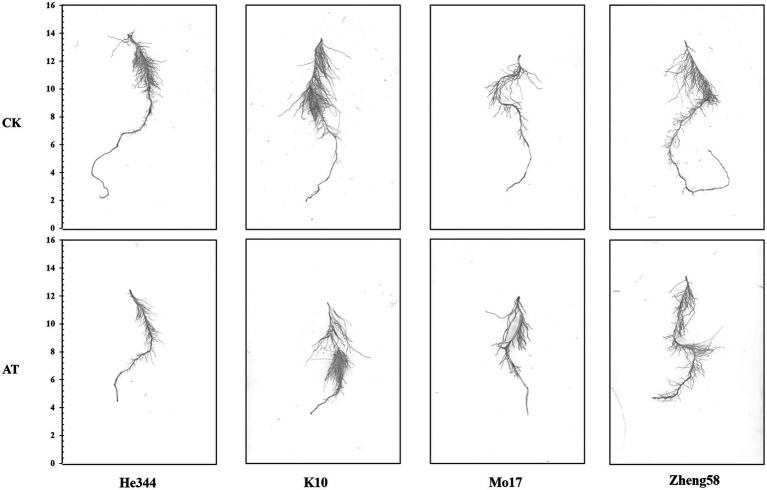
Morphological change in maize under control (CK) and alkaline (AT) treatment. Comparison of roots of maize inbred line He344; inbred line k10; inbred line Mo17; and inbred line Zheng58 under control and alkaline treatment for 10 days. The alkali-sensitive inbred lines Mo17 and He344 are from the Lancaster group, and the alkali-tolerant inbred lines Zheng58 and K10 are from the PA group.

The alkaline tolerance of 200 inbred lines at the seedling stage was evaluated (Figure 1). The descriptive statistics for alkaline resistant traits RSL, RSFW, RRFW, RSDW, RRDW, RRL, RRAD, RRSA, RRV, and RRTN evaluated in an artificial environment are presented in Table 1. Under both the control and alkaline conditions, the indicators of alkali tolerance were determined, and descriptive statistics were executed for the related characters, including the determination of the maximum value, minimum value, and standard deviation (SD). For these traits, there were abundant phenotypic variations, they ranged from 0.5487 (RSFW) to 1.0847 (RRAD). The RRL varied from 0.2908 to 0.9935, the RRTN varied from 0.1959 to 0.9916, the RSDW varied from 0.2846 to 0.9972, the RSL varied from 0.4709 to 0.9830, the RSFW varied from 0.1857 to 0.9399, the RRFW varied from 0.4069 to 0.9958, the RRDW varied from 0.4448 to 0.9957, the RRSA varied from 0.3679 to 0.9986, the RRV varied from 0.4152 to 0.9971, and the RRAD varied from 0.8180 to 1.4405. Curiously, the RRAD of some inbred lines had a value of >1.000, suggesting that the relative root average diameter of these inbred lines was actually stimulated by the alkali treatment. In general, the 10 traits analyzed in the panel fit a normal distribution. ANOVA tests showed statistically significant genotypic differences for the 10 relative traits. In the association panel, the broad-sense heritability (H^2^) of the 10 relative traits ranged from 24.00% (RRAD) to 67.30% (RRTN; Supplementary Table S3).

**Table 1:** Descriptive statistics of seedling stage traits under alkaline and normal conditions.

Trait	*n*	Mean	Maximum	Minimum	SD	Kurtosis	Skewness
RSL	200	0.7277	0.9830	0.4709	0.1013	−0.4045	0.0909
RSFW	200	0.5487	0.9399	0.1857	0.1476	−0.5848	0.1255
RRFW	200	0.7641	0.9958	0.4069	0.1270	−0.5716	0.0524
RSDW	200	0.6412	0.9972	0.2846	0.1524	−0.3523	−0.5156
RRDW	200	0.8274	0.9957	0.4448	0.1104	0.0542	−0.6983
RRL	200	0.6664	0.9935	0.2908	0.1610	−0.7076	−0.1055
RRAD	200	1.0847	1.4405	0.8180	0.1030	0.1840	0.2458
RRSA	200	0.7116	0.9986	0.3679	0.1438	−0.8569	−0.0881
RRV	200	0.7448	0.9971	0.4152	0.1405	−0.8175	−0.2192
RRTN	200	0.6806	0.9916	0.1959	0.1876	−0.7629	−0.2837

The traits of 200 inbred lines were evaluated at the seedling stage under normal and alkaline conditions (25 mmol/LNa_2_CO_3_). Ten relative traits were obtained by dividing the directly measured trait values under alkaline conditions (RL, RAD, RSA, RRV, RTN, SDW, RDW, SFW, and RFW) by the corresponding values under normal conditions.

**Figure 2:**
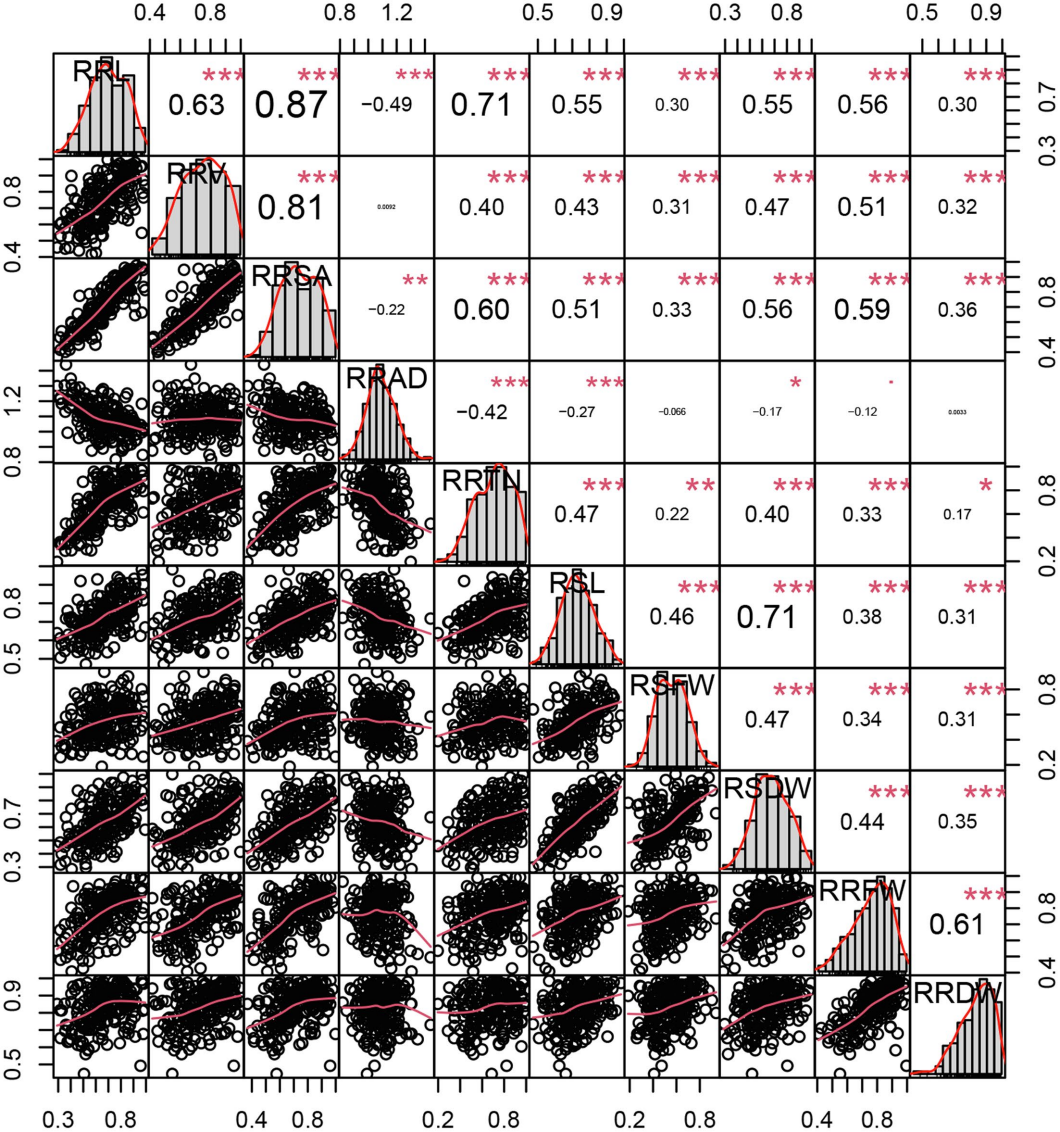
Distributions of and correlations between 10 relative phenotypic traits. The frequency distribution histograms of 10 traits are located on the diagonal line, the area below the diagonal line is the scatter plot of the traits, and the area above is the correlation coefficient between each pair of traits. ^*^, ^**^, and ^***^ indicate significance at *p* < 0.05, *p* < 0.01, and *p* < 0.001, respectively.

In total, 10 traits were measured (Figure 2) and their correlations with each other were determined using the relative value of each trait under alkali treatment. The traits analyzed at the seedling stage characterized maize growth, indicating that these ten traits can be used as indicators of maize alkali tolerance at the seedling stage. Except for in the case of RRV and RRAD, RSFW and RRAD, RRDW and RRAD, RRAD and the other traits had a significant negative correlation (*p* < 0.05), such that RRL and RRAD were −0.49, the RRTN and RRAD were −0.42, and the RSL and RRAD were −0.27 (*p* < 0.001). Except for the RRAD, significant positive correlations were achieved between the other traits (*r* = 0.17–0.87, *p* < 0.05). The three traits that were most significantly associated were RRL and RRSA (0.87), RRV and RRSA (0.81), RRL and RRTN (0.71), and RSL and RSDW (0.71; *p* < 0.001; Supplementary Table S4). The 10 traits were approximated as a normal distribution (kurtosis and skewness< ±1), and showed a significant correlation, indicating the suitability of the 10 traits for further GWAS analysis.

### Population structure and GWAS analysis

**Figure 3:**
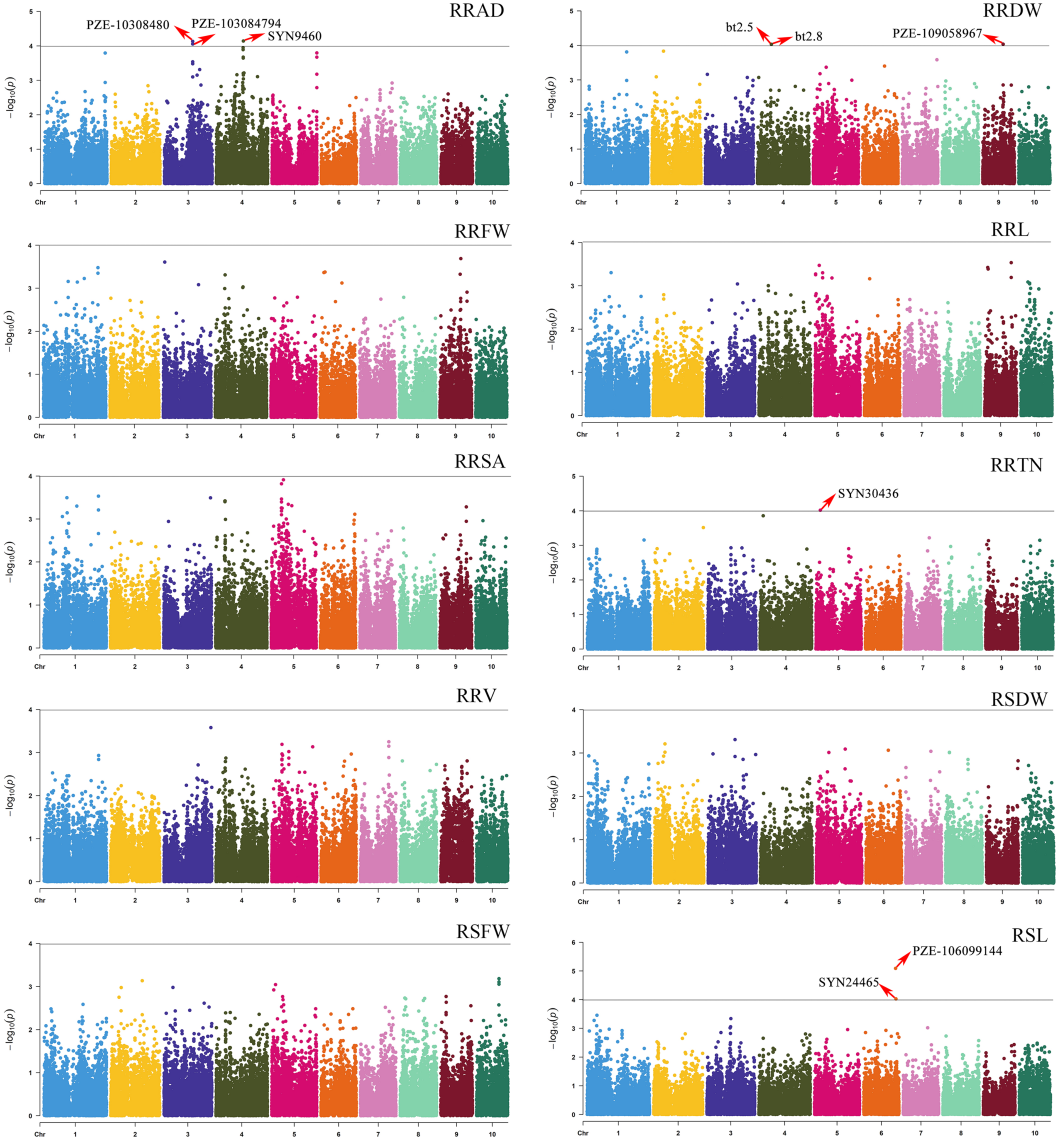
Manhattan plots of GWAS results showing the significant SNPs associated with 10 relative phenotypic traits. The traits included the following: the relative root average diameter (RRAD), relative root fresh weight (RRFW), relative root surface area (RRSA), relative root volume (RRV), relative shoot fresh weight (RSFW), relative root dry weight (RRDW), relative root length (RRL), relative root tip number (RRTN), relative shoot dry weight (RSDW), and relative seeding length (RSL).

The population structure was calculated using STRUCTURE version 2.3. When K = 6, the 200 inbred lines could be grouped into six large subgroups (Supplementary Figure S1). GWAS was used for the 10 relative traits (RSL, RSFW, RRFW, RSDW, RRDW, RRL, RRAD, RRSA, RRV, and RRTN) for the 200 maize inbred lines. GWAS of the SNP markers and related traits was performed using TASSEL 5.0 software, using a mixed linear model (MLM) combined with population structure and kinship. From this analysis, nine SNPs were shown to be significantly associated with four traits (the *p* values ranged from 8.1081E-06 to 9.6125E-05; Figure 3).

Among the nine associated SNPs, the distance between bt2.5 and bt2.8 on chromosome 4 was only 706 bp (for RRDW), and the distance between PZE-103084802 and PZE-103084794 on chromosome 3 was only 42 kb (for RRAD). These SNPs were located on maize chromosomes 3, 4, 5, 6, and 9, with the highest number of SNPs (3) on chromosome 4. A single SNP associated with RRT was discovered. Two SNPs were detected for RSL, and the most significantly associated SNP was for PZE-106099144 (*p* = 8.1081E-06). The three SNPs were significantly associated with RRAD and were located on chromosomes 4 (SYN9460, *p* = 7.21E-05), 3 (PZE-103084802, *p* = 7.45E-05), and 3 (PZE-103084794, *p* = 8.83E-05). The three SNPs were significantly associated with RRDW and were located on chromosomes 9 (PZE-109058967, *p* = 9.18E-05), 4 (bt2.5, *p* = 9.28E-05), and 4 (bt2.8, *p* = 9.28E-05). Four relative traits containing 9 SNPs explained 7.94%–10.71% of the total phenotypic variation (Table 2).

**Table 2:** SNPs associated with alkaline tolerance detected in 200 maize inbred lines.

Trait	SNP	Alleles	Chromosome	Position	*p-value*	*R^2^*
RSL	PZE-106099144	A/C/G/T	6	152,684,808	8.11E-06	10.5%
RRAD	SYN9460	A/G	4	129,386,027	7.21E-05	10.7%
RRAD	PZE-103084802	A/C/T	3	136,692,058	7.45E-05	8.7%
RRAD	PZE-103084794	C/G/T	3	136,649,558	8.83E-05	8.7%
RRDW	PZE-109058967	A/G	9	97,545,021	9.18E-05	8.4%
RRDW	bt2.5	A/G	4	66,290,049	9.28E-05	8.3%
RRDW	bt2.8	A/G	4	66,290,755	9.28E-05	8.3%
RSL	SYN24465	C/G/T	6	154,387,810	9.35E-05	7.9%
RRTN	SYN30436	A/G	5	20,797,115	9.61E-05	9.3%

The location is indicated by the chromosome and base pair position. Values of *p* less than 10^−4^ corresponding to a 5% type I error are displayed in scientific notation. *R*^2^: the percentage of phenotypic variation.

Among the nine SNPs found for the four traits, nine candidate genes that were directly closest to the physical location of the SNPs were mined using the B73 RefGen_v4 Maize Gene Database (Table 3). Two genes were found to be associated with RSL (*Zm00001d038265*, and *Zm00001d038320*). Three genes were found to be associated with RRAD (*Zm00001d050905*, *Zm00001d041766*, and *Zm00001d041767*). Three genes were found to be associated with RRDW (*Zm00001d046591*, *Zm00001d050109*, and *Zm00001d050110*). For RRTN, only one candidate gene was associated (*Zm00001d013802*). Furthermore, when the LD decay distance was changed to r^2^ = 0.2, an additional 200 candidate genes were added to the 9 associated SNPs (Supplementary Table S5). They were distributed on chromosomes 1–10: the PZE-106099144 (SL) locus contained 45 genes, the SYN24465 (SL) locus contained 42 genes, the SYN9460 (RAD) locus contained 10 genes, the PZE-103084802 (RAD) locus contained 17 genes, the PZE-103084794 (RAD) locus contained 19 genes, the PZE-109058967 (RDW) locus contained 36 genes, the bt2.5 (RDW) locus contained 9 genes, the bt2.8 (RDW) locus contained 9 genes, and the SYN30436 (RRTN) locus contained 39 genes.

**Table 3:** Functions of candidate genes that are directly associated with alkaline tolerance traits.

Trait	SNP	Gene ID	Gene function
RSL	PZE-106099144	*Zm00001d038265*	uncharacterized
RRAD	SYN9460	*Zm00001d050905*	dynein light chain type 1 family protein
RRAD	PZE-103084802	*Zm00001d041767*	PHD finger protein
RRAD	PZE-103084794	*Zm00001d041766*	transcription initiation factor TFIID subunit 8
RRDW	PZE-109058967	*Zm00001d046591*	vacuolar-type H^+^-pyrophosphatase5
RRDW	bt2.5	*Zm00001d050109*	water channel
RRDW	bt2.8	*Zm00001d050110*	aspartic proteinase nepenthesin-1
RSL	SYN24465	*Zm00001d038320*	ethylene-responsive transcription factor ABR1
RRTN	SYN30436	*Zm00001d013802*	uncharacterized

### RNA-seq analysis of leaves transcripts in response to alkali stress

**Figure 4:**
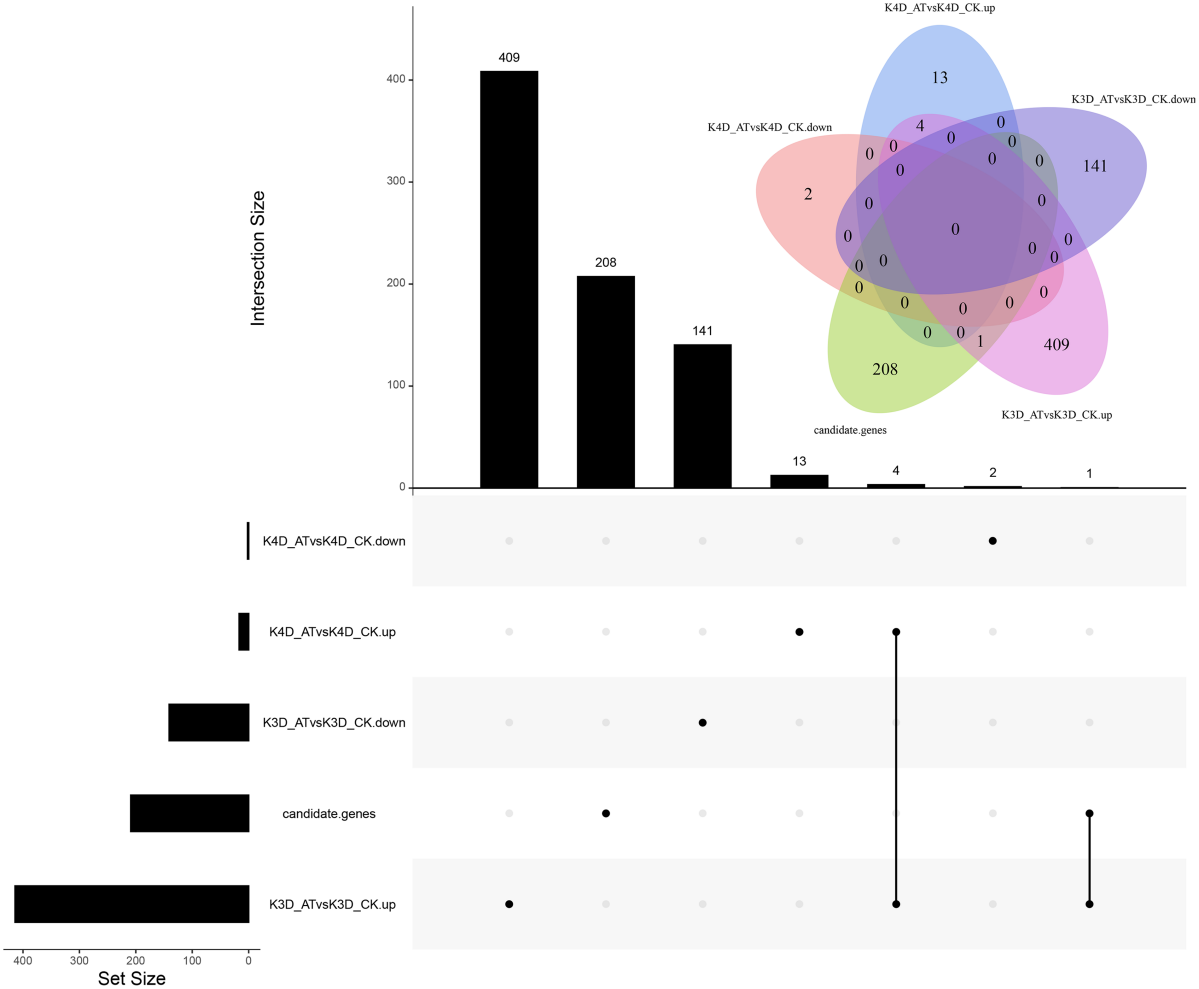
Venn diagram of DEGs distributed in maize inbred lines K10. The treatments included the normal control treatment for 3 and 4 days (CK), and the alkaline treatment for 3 and 4 days (AT). The alkaline treatment for 3 and 4 days are labeled “K3D_AT”and “K4D_AT,” respectively. The five treatment-line comparisons groups included the following: alkaline treatment for 3 days vs. normal control treatment for 3 days, upregulated expression (K3D_ATvsK3D_CK.up); alkaline treatment for 3 days vs. normal control treatment for 3 days, downregulated expression (K3D_ATvsK3D_CK.down); alkaline treatment for 4 days vs. normal control treatment for 4 days, upregulated expression (K4D_ATvsK4D_CK.up); alkaline treatment for 4 days vs. normal control treatment for 4 days, downregulated expression (K4D_ATvsK4D_CK.down); and candidate genes.

To help identify candidate genes, the maize inbred line K10 was selected as an alkali stress tolerant example and its genome-wide gene expression levels were analyzed using RNA-seq. For downregulated DEGs, two were identified in the K4D_ATvsK4D_CK comparison group and 141 DEGs were identified in the K3D_ATvsK3D_CK comparison group. For the upregulated DEGS (|log2FC| ≥ 0.585, *p* < 0.05), 17 were identified in the K4D_ATvsK4D_CK comparison group, 414 were identified in the K3D_ATvsK3D_CK comparison group, and four were identified in the K4D_ATvsK4D_CK and K3D_ATvsK3D_CK comparison groups (Figure 4).

A total of 209 candidate genes were associated with SNPs, and one important DEG was identified, including one candidate gene (*Zm00001d038250*) that was only upregulated in the K3D_ATvsK3D_CK comparison group. Four DEGs were upregulated in the K3D_ATvsK3D_CK and K4D_ATvsK4D_CK comparison group, including four candidate genes (*Zm00001d027619*, *Zm00001d032973*, *Zm00001d001820*, and *Zm00001d001960*). Putative protein functions are listed in Table 4, determined by NCBI. *Zm00001d038250* encoded a HSP40/DNAJ peptide-binding protein, *Zm00001d027619* encoded a beta-amylase, *Zm00001d032973* encoded a glycerol-3-phosphate acyltransferase7, *Zm00001d001820* encoded a protochlorophyllide reductase 1, and *Zm00001d001960* encoded a flavanone 3-dioxygenase 1.

**Table 4:** Candidate DEGs that are consistent between the K4D_ATvsK4D_CK and K3D_ATvsK3D_CK comparison groups.

Gene ID	K4D_ATvsK4D_CK	K3D_ATvsK3D_CK	Gene function	*Arabidopsis* best hit	Rice best hit
*Zm00001d038250*	no	up	HSP40/DNAJ peptide-binding protein	DNAJ heat shock family protein	DNAJ protein homolog 1
*Zm00001d027619*	up	up	beta-amylase	Glycoside hydrolase family 14	beta-amylase 2
*Zm00001d032973*	up	up	glycerol-3-phosphate acyltransferase7	glycerol-3-phosphate acyltransferase 6	glycerol-3-phosphate 2-O-acyltransferase 6
*Zm00001d001820*	up	up	protochlorophyllide reductase 1	protochlorophyllide oxidoreductase A	protochlorophyllide reductase A
*Zm00001d001960*	up	up	Flavanone 3-dioxygenase 1	flavanone 3-hydroxylase	flavanone 3-dioxygenase 1-like

### Functional prediction of candidate genes by GWAS and RNA-seq analysis

**Figure 5:**
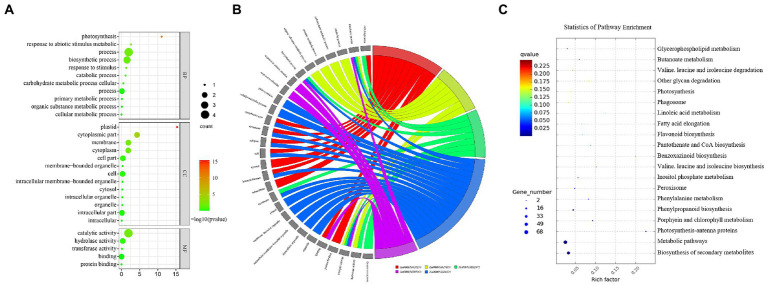
GO annotations and KEGG pathways of DEGs. **(A)** Bubble chart of GO classifications of five DEGs. **(B)** Chord chart of GO annotation corresponding to five DEGs. **(C)** The red box is the KEGG pathway map of Zm00001d02761, Zm00001d001820, and Zm00001d001960 genes.

There were a total of 37 GO terms associated with the five candidate genes, three candidate genes were associated with four KEGG pathways, and one KEGG pathway (zma01110) was co-enriched by the three genes (Tables 5, 6). These GO terms were functionally related to three broad categories. The first function described was cellular components, including cytoplasmic part (*Zm00001d001820* and *Zm00001d038250*), cytoplasm (*Zm00001d001820* and *Zm00001d038250*), cell part (*Zm00001d038250* and *Zm00001d001820*), cell (*Zm00001d001820* and *Zm00001d038250*), cytosol (*Zm00001d038250*), intracellular part (*Zm00001d038250* and *Zm00001d001820*), intracellular (*Zm00001d038250*), membrane (*Zm00001d032973* and *Zm00001d001820*), plastid (*Zm00001d001820*), membrane-bounded organelle (*Zm00001d001820*), intracellular membrane-bounded organelle (*Zm00001d001820*), intracellular organelle (*Zm00001d001820*), and organelle (*Zm00001d001820*). The second type described was biological processes, which involved cellular process (*Zm00001d038250* and *Zm00001d032973*), metabolic process (*Zm00001d027619*, *Zm00001d032973*, *Zm00001d001820*, and *Zm00001d001960*), catabolic process (*Zm00001d027619*), carbohydrate metabolic process (*Zm00001d027619*), primary metabolic process (*Zm00001d027619*), organic substance metabolic process (*Zm00001d027619*), biosynthetic process (*Zm00001d032973*, *Zm00001d001820* and *Zm00001d001960*), response to abiotic stimulus (*Zm00001d00196*0), response to stimulus (*Zm00001d001960*), photosynthesis (*Zm00001d001820*), and cellular metabolic process (*Zm00001d001820*). The third type of function described was molecular functions, which involved binding (*Zm00001d001960* and *Zm00001d038250*), protein binding (*Zm00001d038250*), catalytic activity (Zm00001d027619, Zm00001d032973, Zm00001d001820, and Zm00001d001960), hydrolase activity (*Zm00001d027619* and Z*m00001d032973*), and transferase activity (*Zm00001d032973*; Table 5; Figures 5A,B). Two major classes of KEGG pathways were identified: the flavonoid synthesis pathway and the metabolic pathway, including starch and sucrose metabolism, and porphyrin and chlorophyll metabolism (Table 6; Figure 5C).

**Table 5:** GO analysis of candidate genes related to the RNA-seq result.

Gene ID	GO
*Zm00001d038250*	GO:0044444; GO:0008150; GO:0005737; GO:0005575; GO:0044464; GO:0005623; GO:0005829; GO:0003674; GO:0044424; GO:0009987; GO:0005622; GO:0005488; GO:0005515
*Zm00001d027619*	GO:0008150; GO:0008152; GO:0016787; GO:0003674; GO:0044238; GO:0071704
*Zm00001d032973*	GO:0008150; GO:0008152; GO:0003824; GO:0016020; GO:0009058; GO:0005575; GO:0016787; GO:0003674; GO:0016740; GO:0009987
*Zm00001d001820*	GO:0009536; GO:0015979; GO:0044444; GO:0008150; GO:0008152; GO:0003824; GO:0005737; GO:0009058; GO:0005575; GO:0044464; GO:0043227; GO:0005623; GO:0043231; GO:0043229; GO:0043226; GO:0003674; GO:0044424; GO:0005622; GO:0044237
*Zm00001d001960*	GO:0009628; GO:0008150; GO:0008152; GO:0003824; GO:0009058; GO:0050896; GO:0003674; GO:0005488

**Table 6:** Distribution of genes and pathways related to the RNA-seq results.

Pathway ID	KEGG term	Gene ID
zma01110	Metabolic pathways	*Zm00001d027619* *Zm00001d001820* *Zm00001d001960*
zma00500	Starch and sucrose metabolism	*Zm00001d027619*
zma00860	Porphyrin and chlorophyll metabolism	*Zm00001d001820*
zma00941	Flavonoid biosynthesis	*Zm00001d001960*

### qRT-PCR validation for differentially expressed genes

**Figure 6:**
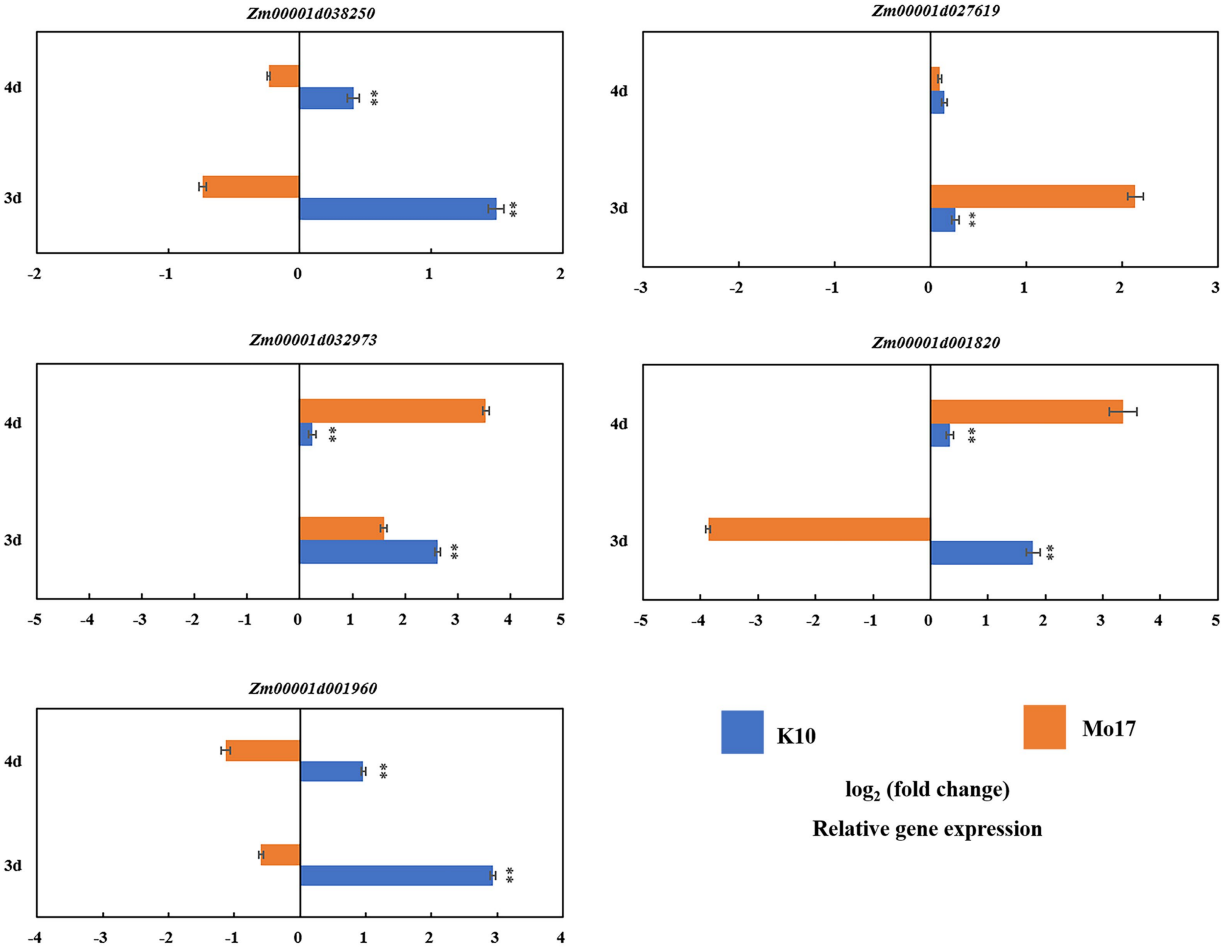
qRT-PCR validation of the GWAS and RNA-seq results. Expression of five candidate genes in alkaline-tolerant inbred line K10 and the alkaline-sensitive inbred line Mo17. Expression analysis was conducted on leaf that were collected at 3 and 4 days under normal control treatment and alkaline treatment, respectively. ^**^ indicate significance at *p* < 0.01.

Five candidate genes were selected based on RNA-seq (|log2FC| ≥ 0.585, *p* < 0.05) for qRT-PCR analysis: *Zm00001d038250, Zm00001d027619*, *Zm00001d032973*, *Zm00001d001820*, and *Zm00001d001960.* When alkali treated for 3 days, the expression level of one gene (*Zm00001d038250*) in K10 was significantly higher (1.49) than that in Mo17 (−0.74) (*p* < 0.01). The expression level of this gene showed a downward trend after 4 days of treatment, but the expression level in B73 (0.410) was still higher than that in Mo17 (−0.237). The expression level of gene *Zm00001d027619* in K10 (0.263) was significantly lower than that in Mo17 (2.140) when treated for 3 days, and there was no significant difference when treated for 4 days. The expression levels of the other two genes (*Zm00001d032973* and *Zm00001d001820*,) in K10 (2.617 and 1.787) were significantly higher than those in Mo17 (1.610 and −3.867) when treated with alkali for 3 days but showed the opposite pattern when treated for 4 days: the expression level in Mo17 (3.533 and 3.353) was significantly higher than that in K10 (0.233 and 0.337). The expression level of the last gene (Zm00001d001960) in K10 (2.933 and 0.960) was significantly higher than that of Mo17 (−0.593 and −1.130) at 3 and 4 days of alkali treatment (Figure 6). The results of RNA-seq and qRT-PCR analysis showed that the expression trends of the five genes were consistent: the expressions were all upregulated after 3 or 4 days of alkaline treatment.

Two candidate genes were shown to be more attractive than the others: *Zm00001d038250* and *Zm00001d001960*, as they were upregulated in K10 and downregulated in Mo17 under both the treatment durations (3 or 4 days). Gene function annotation suggested that they may be involved in alkaline tolerance-related functions, such as heat shock proteins and flavonoid metabolic pathways.

## Discussion

Maize is an alkali-sensitive crop that is grown on salt-contaminated soil due to soil salinization (Luo et al., 2021). Soil salinization ultimately affects maize yield, but the effect of salinization at the seedling stage is reflected in traits such as seedling length and root fresh weight. In previous studies, the SL, SFW, RFW, SDW, and RDW were used to assess the salinity tolerance of maize, highlighting that these traits showed a significant positive correlation to salinity tolerance (Cui et al., 2014; Luo et al., 2019, 2021). In addition to using the above traits, traits such as RL and RAD were added in this study. A total of 10 relative traits were determined at the seedling stage for GWAS. Among the 200 maize inbred lines that were studied, a large range of phenotypic variation was observed. Except for RAD, which was significantly negatively correlated with other traits, the remaining traits showed significant positive correlations that were consistent with previous studies. The newly added trait, RL, was also significantly positively correlated with other traits but extremely significantly negatively correlated with RRAD (r^2^ = −0.49, *p* < 0.001). The mean RRAD of 200 inbred lines was 1.0847, and the average RRL of 200 inbred lines was 0.6664. This indicated that maize may resist external alkali stress by thickening and shortening its roots.

GWAS is now commonly used to detect complex trait variants and predict candidate genes. However, there are still problems with false positives using this method (Wang et al., 2019). RNA-seq has emerged as an important tool for analyzing genome-wide expression patterns (Tai et al., 2016). Nevertheless, potential candidate genes are difficult to identify from the large number of DEGs obtained by RNA-seq. In recent years, GWAS combined in combination with RNA-seq has been used to detect novel genes associated with complex traits in crops. For example, five candidate genes potentially involved in maize root development were predicted using GWAS in combination with RNA-seq. Furthermore, ten candidate genes related to low-temperature tolerance were also identified (Wang et al., 2019; Zhang et al., 2020). This study identified five candidate genes (*Zm00001d038250*, *Zm00001d027619*, *Zm00001d032973*, *Zm00001d001820*, and *Zm00001d001960*) associated with alkali tolerance through this method. Among the identified genes, *Zm00001d038250* and *Zm00001d001960* had significant differences in expression between the sensitive and tolerant maize lines, may provide valuable genetic information on the mechanisms of alkali tolerance in maize at the seedling stage.

To assess the reliability of the SNPs in this study, the nine SNPs obtained from this research were compared with previously identified and published QTLs and SNPs. Three of the loci overlapped with the physical locations of previously published QTLs. SYN30436 on chromosome 5 was located in proline-associated *qPC-5-1* under alkaline treatment (Zhang et al., 2018). Two SNPs located on chromosome 3 were located on QTLs associated with shoot dry weight (Luo et al., 2019). The parallel between the examples that have been previously described and the examples identified in this study proved that the SNPs of the alkali tolerance-related traits in this study were reliable for maize breeding. The contribution rate of SNPs in this study was 8.4%–10.7%, which may be because root-related traits are controlled by many small-effect QTLs (Burton et al., 2014; Li et al., 2015).

*Zm00001d038250* encoded the DNAJ protein, also known as HSP40, which is a member of the conserved co-chaperone protein family. It is homologous to rice *LOC_ OS05G0427900* and *Arabidopsis AT1G44160*. As a co-chaperone of HSP70, the DNAJ protein can promote the ATPase activity of HSP70 under stress conditions and participate in the maintenance of intracellular protein folding, complex depolymerization, and other vital activities (Rajan and D’Silva, 2009; Chen et al., 2014). It was found that *Zm00001d027619* in maize encoded β-amylase protein and contains an AmyAc conserved domain. However, little research has been presented on β-amylase and its response to abiotic stress using unstructured carbon composed of soluble sugars and starches (Dickman et al., 2019; Zhen et al., 2020).

Next, *Zm00001d032973* encoded a glycerol-3-phosphate acyltransferase protein (GPAT). Three types of GPATs have been found in plant cells, which are located in the plastids (including chloroplasts), endoplasmic reticulum, and mitochondria (Murata and Tasaka, 1997; Zheng et al., 2003; Gidda et al., 2009). Among these, GPATs in plastids/chloroplasts are soluble proteins, which affect plants’ cold tolerance by affecting the saturation of chloroplast membrane lipid molecules (Murata and Tasaka, 1997; Yokoi et al., 1998; Zhu et al., 2009; Sui et al., 2017). GPATs distributed in the endoplasmic reticulum and mitochondria are membrane-bound proteins, which participate in the biosynthesis of triacylglycerol (TAGs) in seeds, thereby affecting the accumulation of seed oil (Zheng et al., 2003). Another gene, *Zm00001d001820*, encoded a protochlorophyllide reductase1 protein (pcr1). A homologous gene in *Arabidopsis thaliana*, *AT5G54190*, was shown to be involved in NYEs/SGRs-mediated chlorophyll degradation for detoxification during seed maturation (Li et al., 2017b). The last gene identified in this study, *Zm00001d001960,* encoded flavanone 3-hydroxylase 1 protein (fht1). It is homologous to rice *LOC_ OS04G0662600* and *Arabidopsis AT3G51240*. In general, flavanone 3-hydroxylase 1 improved stress tolerance by upregulating its expression under water-related stress and increasing the content of flavonoids (Klimek-Chodacka et al., 2016; Hodaei et al., 2018).

**Figure 7:**
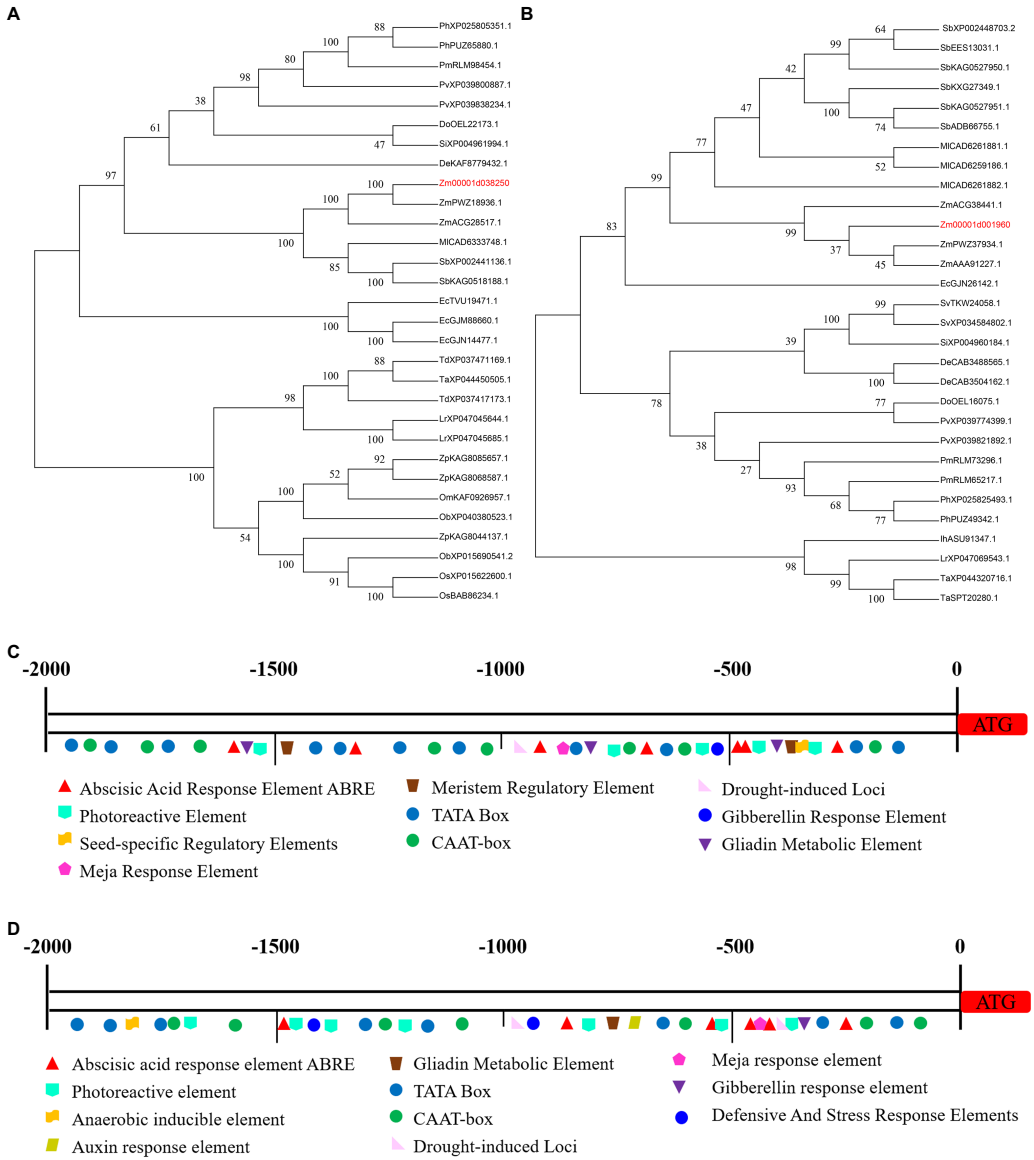
The characterization of candidate genes. **(A)** Evolutionary tree of Zm00001d038250 protein. **(B)** Evolutionary tree of Zm00001d001960 protein. **(C)** The promoter region of *Zm00001d038250* gene. **(D)** The promoter region of *Zm00001d001960* gene.

The two candidate genes (*Zm00001d038250* and *Zm00001d001960*) are thought to function in alkali tolerance, similar to the HSP40 protein and flavanone 3-hydroxylase 1 activity. *Zm00001d038250* encoded the HSP40 protein (sHSP), a heat shock protein (HSP), which is a special protein produced by the body in response to stress. Its structure is highly conserved, and it mainly acts as a molecular chaperone to play biological functions such as folding and transportation (Voellmy and Boellmann, 2007). Maize mitochondrial sHsps improved mitochondrial electron transport during salt stress, mainly by protecting NADH: ubiquinone oxidoreductase activity (Hamilton and Heckathorn, 2001). Overexpression of HSP40 in *Arabidopsis* improved salt tolerance by increasing root length under 120 mM NaCl treatment (Zhao et al., 2010). The protein BIL2 in the *Arabidopsis* HSP40 family induced cell elongation during BR signaling by promoting ATP synthesis in mitochondria, thereby increasing inflorescence root length in response to salt and light stress (Bekh-Ochir et al., 2013). Zm00001d038250 and ZmPWZ18936.1 proteins (NCBI accession number: PWZ18936.1) are evolutionarily on the same branch, and may have some similarities in function. The *Zm00001d038250* gene promoter region contained not only CAAT-box core elements but also hormone-, light-, defense-, metabolism-, and stress-related elements (Figures 7A,C; Supplementary Table S6).

*Zm00001d001960* putatively encoded flavanone 3-hydroxylase 1(F3H). Flavonoids are a group of substances with various phenolic structures, which participate in anti-oxidative stress processes, and also participate in some biotic and abiotic stress response processes (Winket-Shirley, 2002; Panche et al., 2016). Researchers have found that recombinant or overexpression of F3H in different crops could modulate naringenin to alleviate growth inhibition and enhance tolerance to salt stress (Li et al., 2017a; Si et al., 2022). Through proteomic analysis of beet seedlings treated with 50 mM NaCl, 135 differentially expressed proteins, including F3H, were mined (Wu et al., 2018). In addition, it was found that under the combined salt and heat stress in rice, overexpression of F3H increased the kaempferol and quercetin content, and then scavenged reactive oxygen species. The content of heat stress transcription factors (HSFs) and heat shock proteins (HSPs) also increased significantly to improve tolerance (Jan et al., 2021). *Zm00001d001960* is involved in metabolic pathway and flavonoid biosynthesis pathway, which may regulate alkali tolerance through this pathway. The Zm00001d001960 protein is evolutionarily in the same large clade with the ZmPWZ37934.1 (NCBI accession number: PWZ37934.1) protein and the ZmAAA91227.1 (NCBI accession number: AAA91227.1) protein, but its credibility is low, it may be that there is only some similarity in their structure. The *Zm00001d001960* gene promoter region contained not only CAAT-box core elements but also hormone-, light-, and metabolism-related elements. (Figures 7B,D; Supplementary Table S7).

In conclusion, the *Zm00001d038250* and *Zm00001d001960* genes were significantly and differentially expressed in tolerant materials under alkaline conditions based on functional analysis and GO enrichment analysis. It was hypothesized that the HSP40 protein encoded by *Zm00001d038250* could limit the ion transport mode, and the F3H protein encoded by *Zm00001d001960* could improve the antioxidant capacity by metabolizing flavonoids, which, in turn, could affect the alkali tolerance of maize seedlings, though this requires further research. Therefore, these genes are attractive genes for the future study of alkali tolerance in maize seedling stage.

## Conclusion

In a population composed of 200 maize inbred lines, we detected 10 alkali tolerance-related traits at the seedling stage and found nine SNPs through GWAS. These SNPs were all related to alkali tolerance during the seedling stage. Through RNA-seq analysis and qPRT-PCR verification, we found that two candidate genes, *Zm00001d038250* and *Zm00001d001960*, were differentially expressed under alkaline treatment. This study provides a genetic basis for molecular-assisted breeding of alkali-tolerant maize seedlings.

## Data availability statement

The datasets presented in this study can be found in online repositories. The names of the repository/repositories and accession number(s) can be found at: https://www.ncbi.nlm.nih.gov/, PRJNA846593.

## Author contributions

ZW and HD: conception or design of the work. CL, YJ, and LL: performed the experiment. RZ, MC, and YZ: analyzed the data. CL and YJ: wrote the manuscript. All authors contributed to the article and approved the submitted version.

## Funding

This research was supported by the National Natural Science Foundation of China (32072128) and Genetically Modified Organisms Breeding Major Projects of China (2016ZX08003003).

## Conflict of interest

The authors declare that the research was conducted in the absence of any commercial or financial relationships that could be construed as a potential conflict of interest.

## Publisher’s note

All claims expressed in this article are solely those of the authors and do not necessarily represent those of their affiliated organizations, or those of the publisher, the editors and the reviewers. Any product that may be evaluated in this article, or claim that may be made by its manufacturer, is not guaranteed or endorsed by the publisher.

## Footnotes

^1^ https://www.ibm.com/support/pages/node/230551

^2^ http://www.maizegdb.org/

^3^ https://www.ncbi.nlm.nih.gov/data-hub/genome/GCA_000005005.6/

^4^ http://www.geneontology.org/

^5^ https://phytozome-next.jgi.doe.gov/

^6^ http://www.genome.ad.jp/kegg/kegg2.html

^7^ http://www.ncbi.nlm.nih.gov/

^8^ http://www.bioinformatics.com.cn

## Supplementary materials

The Supplementary Materials for this article can be found online at: https://www.frontiersin.org/articles/10.3389/fpls.2022.963874/full#supplementary-material
