# Supplementary material for: GWAS and RNA-seq analysis uncover candidate genes associated with alkaline stress tolerance in maize (Zea mays L.) seedlings
Source: Front Plant Sci. 2022 Jul 18;13:963874. doi: 10.3389/fpls.2022.963874 (PMC9340071; doi:10.3389/fpls.2022.963874)

**Supplementary file 5：**

**Figure S1:** Estimated population structure of the 200 maize inbred lines with values of K = 6.


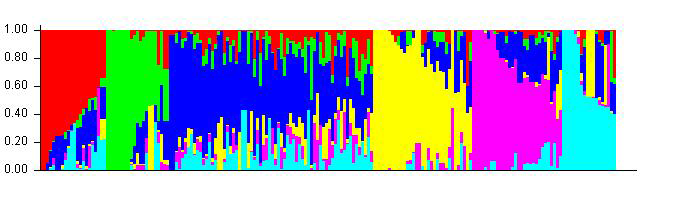

Supplement: Supplementary file 1 [file Data_Sheet_1.zip › Figure s1.docx]
