# Supplementary material for: GWAS and RNA-seq analysis uncover candidate genes associated with alkaline stress tolerance in maize (Zea mays L.) seedlings
Source: Front Plant Sci. 2022 Jul 18;13:963874. doi: 10.3389/fpls.2022.963874 (PMC9340071; doi:10.3389/fpls.2022.963874)
Supplement: Supplementary file 1 [file Data_Sheet_1.zip › Table s1.docx]

**Supplementary file 1：**

**Table S1:** 200 maize inbred lines

| No. | Name | No. | Name | No. | Name |
| --- | --- | --- | --- | --- | --- |
| 1 | Jinhuang96B | 68 | H21 | 135 | Dong91 |
| 2 | Zhongzi01 | 69 | Jing7 | 136 | Hai014 |
| 3 | Shen118 | 70 | Huangyesi | 137 | Zhonghuang64 |
| 4 | Zao23 | 71 | K12 | 138 | CA112 |
| 5 | Han21 | 72 | Suixi605 | 139 | Ji818 |
| 6 | SH15 | 73 | 502 | 140 | Qi319 |
| 7 | Chuan273 | 74 | 515 | 141 | Dian11 |
| 8 | Chuan321 | 75 | Dhuang212 | 142 | 888-9 |
| 9 | Y7 | 76 | Ji35 | 143 | WF9 |
| 10 | Jinhuang59 | 77 | LX9801 | 144 | Ying64 |
| 11 | 31778 | 78 | Huang428-3 | 145 | Xinzi153-2 |
| 12 | Jinhuang55 | 79 | Si287 | 146 | Chong72 |
| 13 | Dan988 | 80 | Si-279 | 147 | Si273 |
| 14 | Shen3336 | 81 | HuangC | 148 | Liao540 |
| 15 | Dan599 | 82 | CAL70 | 149 | Liao184 |
| 16 | Nongda178 | 83 | K14 | 150 | Liao9586 |
| 17 | P138 | 84 | 7167-1 | 151 | Suixi707 |
| 18 | Dan3130 | 85 | 7537-1 | 152 | Dan360 |
| 19 | Yu12 | 86 | PI10 | 153 | Dan340 |
| 20 | Zhonghuang204 R2040 | 87 | Zheng29 | 154 | Dan598 |
| 21 | Ji412 | 88 | 501 | 155 | Tie9010 |
| 22 | Ji419 | 89 | S7913 | 156 | Zheng22 |
| 23 | Ji465 | 90 | CA156 | 157 | DH34 |
| 24 | Ji842 | 91 | 7165-1 | 158 | Moqun17 |
| 25 | CA091 | 92 | N528-1(1284) | 159 | B73 |
| 26 | J002 | 93 | Zheng30 | 160 | U8112 |
| 27 | 77 | 94 | 653 | 161 | B84 |
| 28 | Zao49 | 95 | Zheng35 | 162 | Ye52106 |
| 29 | Liaobai371 | 96 | B234 | 163 | Ji81162 |
| 30 | Huotanghuang | 97 | Ji046 | 164 | 7884 |
| 31 | Guan17 | 98 | Shen5003 | 165 | W24 |
| 32 | 706fu | 99 | 7922 | 166 | Si144 |
| 33 | Zhong17 | 100 | Ji4112 | 167 | 5022(B) |
| 34 | Zao8-3（1263） | 101 | 8902 | 168 | Zhonghuang68 |
| 35 | H3 | 102 | 835 | 169 | Linxi11 |
| 36 | 485 | 103 | C8605-2 | 170 | 444 |
| 37 | Si533 | 104 | Dan9046 | 171 | Si387 |
| 38 | Ji992 | 105 | Liao2345 | 172 | B84/C107-7 |
| 39 | C416 | 106 | Liao3053 | 173 | 6523 |
| 40 | D185 | 107 | Liao5114 | 174 | B104 |
| 41 | Jia-034 | 108 | Liao6082 | 175 | 8107 |
| 42 | Mo17 | 109 | 3189 | 176 | 2002F22 |
| 43 | Zac546 | 110 | 8001 | 177 | 32 |
| 44 | 416 | 111 | 803 | 178 | 832 |
| 45 | Dan1324 | 112 | K22 | 179 | 488 |
| 46 | Ji846 | 113 | Zheng58 | 180 | 8129 |
| 47 | Ji477 | 114 | K10 | 181 | 4866 |
| 48 | He344 | 115 | Dong46 | 182 | TS6278 |
| 49 | C649 | 116 | Ji53 | 183 | Jinhuang63 |
| 50 | Si-419 | 117 | Cai11-8 | 184 | Jinhuang73 |
| 51 | Ji495 | 118 | Luyuan92 | 185 | Jinhuang76 |
| 52 | J001 | 119 | Zi330 | 186 | 53xuan3 |
| 53 | Longkang11 | 120 | 8415 | 187 | 812 |
| 54 | D387 | 121 | 81565 | 188 | Qing795 |
| 55 | Ji1037 | 122 | H10 | 189 | 5311 |
| 56 | Suixi701 | 123 | Chang3 | 190 | Q1261 |
| 57 | 5213 | 124 | Zhongzong4c1-3-2-2-b-5-2-1-b | 191 | Zheng28 |
| 58 | Wenhuang | 125 | Zheng28 | 192 | PH4CV |
| 59 | Danhuang02 | 126 | Zong31 | 193 | PH6WC |
| 60 | CN962 | 127 | Zhong451 | 194 | Shuang105 |
| 61 | Luyuan133 | 128 | Dong237 | 195 | PI36 |
| 62 | H152 | 129 | 200B | 196 | CWF  (zhongnuomuS8) |
| 63 | 374 | 130 | Zhong106 | 197 | CWM (zhongnuofuS9) |
| 64 | Tangsipingtou | 131 | CA339 | 198 | Wa138 |
| 65 | Zi495 | 132 | Ji63 | 199 | 5dong |
| 66 | Huangzaosi | 133 | Benm130 | 200 | Cheng351 |
| 67 | Ji853 | 134 | Dong156 |  |  |
