# Supplementary material for: GWAS and RNA-seq analysis uncover candidate genes associated with alkaline stress tolerance in maize (Zea mays L.) seedlings
Source: Front Plant Sci. 2022 Jul 18;13:963874. doi: 10.3389/fpls.2022.963874 (PMC9340071; doi:10.3389/fpls.2022.963874)
Supplement: Supplementary file 1 [file Data_Sheet_1.zip › Table s2.docx]

**Supplementary file 2：**

**Table S2:** Primer sequences used for quantitative real-time PCR.

| **Gene name** | **Forward primer (5'-3')** | **Reverse primer (5'-3')** |
| --- | --- | --- |
| *Zm00001d038250* | GAAAGACTCAGGCAAAGTGGC | TGCAAGCAGAGGCAGCAA |
| *Zm00001d027619* | GCGGCAACGTCGGTGATTCA | GAAGTCGGCGTAGCACTGGATG |
| *Zm00001d032973* | TGAGCATGTTCCACGGCACAAC | TGTCCTTCCTGGTGAGGCTTGT |
| *Zm00001d001820* | TGCTCTTCCCGCCTTTCCAGAA | GAACGACGCCGAGTCCTTGTT |
| *Zm00001d001960* | ATACTGGACCATCCCATCACCTT | GCGAACTCCTTGTTGGCACTCTT |
