# Supplementary material for: GWAS and RNA-seq analysis uncover candidate genes associated with alkaline stress tolerance in maize (Zea mays L.) seedlings
Source: Front Plant Sci. 2022 Jul 18;13:963874. doi: 10.3389/fpls.2022.963874 (PMC9340071; doi:10.3389/fpls.2022.963874)
Supplement: Supplementary file 1 [file Data_Sheet_1.zip › Table s3.docx]

**Supplementary file 3：**

**Table S3:** ANOVA results and heritability of 10 traits of the lines composing the association panel.

| Traits | *F* value | *H^2^*（%） |
| --- | --- | --- |
| RRL | 2.775*** | 37.31 |
| RRV | 2.098*** | 26.96 |
| RRSA | 2.415*** | 31.58 |
| RRAD | 2.284*** | 30.00 |
| RRTN | 7.070*** | 67.30 |
| RSL | 3.298*** | 41.18 |
| RSFW | 4.738*** | 54.84 |
| RRFW | 2.928*** | 37.80 |
| RSDW | 4.208*** | 52.34 |
| RRDW | 1.907*** | 24.00 |

*H*^2^: represented broad-sense heritability. *, ** and *** represent indicate significant level at *P* < 0.05, *P* < 0.01 and *P* < 0.001, respectively.
