# Supplementary material for: GWAS and RNA-seq analysis uncover candidate genes associated with alkaline stress tolerance in maize (Zea mays L.) seedlings
Source: Front Plant Sci. 2022 Jul 18;13:963874. doi: 10.3389/fpls.2022.963874 (PMC9340071; doi:10.3389/fpls.2022.963874)
Supplement: Supplementary file 1 [file Data_Sheet_1.zip › Table s4.docx]

**Supplementary file 4：**

**Table S4:** Correlation coefﬁcients (r) between 10 phenotypic traits of 200 maize inbred lines under alkaline conditions.

| Trait | RRL | RRV | RRSA | RRAD | RRTN | RSL | RSFW | RSDW | RRFW | RRDW |
| --- | --- | --- | --- | --- | --- | --- | --- | --- | --- | --- |
| RRL | 1 |  |  |  |  |  |  |  |  |  |
| RRV | .628*** | 1 |  |  |  |  |  |  |  |  |
| RRSA | .866*** | .814*** | 1 |  |  |  |  |  |  |  |
| RRAD | -.486*** | .139 | -.222** | 1 |  |  |  |  |  |  |
| RRTN | .712*** | .403*** | .603*** | -.423*** | 1 |  |  |  |  |  |
| RSL | .549*** | .427*** | .506*** | -.267*** | .466*** | 1 |  |  |  |  |
| RSFW | .298*** | .311*** | .330*** | -.066 | .219** | .456*** | 1 |  |  |  |
| RSDW | .550*** | .475*** | .556*** | -.165* | .400*** | .707*** | .468*** | 1 |  |  |
| RRFW | .556*** | .511*** | .591*** | -.120* | .330*** | .380*** | .343*** | .443*** | 1 |  |
| RRDW | .300*** | .321*** | .361*** | .003 | .174* | .312*** | .312*** | .347*** | .606*** | 1 |

*, ** and *** indicate significant levels at *P* < 0.05, *P* < 0.01 and *P* < 0.001, respectively.
