# Supplementary material for: GWAS and RNA-seq analysis uncover candidate genes associated with alkaline stress tolerance in maize (Zea mays L.) seedlings
Source: Front Plant Sci. 2022 Jul 18;13:963874. doi: 10.3389/fpls.2022.963874 (PMC9340071; doi:10.3389/fpls.2022.963874)
Supplement: Supplementary file 1 [file Data_Sheet_1.zip › Table s5.docx]

**Supplementary file 6：**

**Table S5:** An additional 200 candidate genes identified within a LD decay(*r^2^*=0.2) of 9 associated SNPs.

| **Trait** | **SNP** | **MaizeGDB ID** | **Function** | **GO** |
| --- | --- | --- | --- | --- |
| RAD | SYN9460 | *Zm00001d050893* | NAC transcription factor | GO:0003677 |
| RAD | SYN9460 | *Zm00001d050894* | ATP-citrate synthase alpha chain protein 3 | GO:0005524 |
| RAD | SYN9460 | *Zm00001d050896* | Uncharacterized | no |
| RAD | SYN9460 | *Zm00001d050897* | MADS-box transcription factor | GO:0003700; GO:0046983; GO:0000977; GO:0045944 |
| RAD | SYN9460 | *Zm00001d050902* | Uncharacterized | no |
| RAD | SYN9460 | *Zm00001d050903* | F-box protein GID2 | GO:0009740; GO:0031146 |
| RAD | SYN9460 | *Zm00001d050904* | Lipid binding protein | no |
| RAD | SYN9460 | *Zm00001d050907* | Uncharacterized | no |
| RAD | SYN9460 | *Zm00001d050908* | OSJNBb0048E02.16-like | no |
| RAD | SYN9460 | *Zm00001d050909* | Uncharacterized | no |
| RRTN | SYN30436 | *Zm00001d013783* | Dof zinc finger protein DOF5.7 | GO:0003677; GO:0003700 |
| RRTN | SYN30436 | *Zm00001d013784* | Uncharacterized | no |
| RRTN | SYN30436 | *Zm00001d013785* | Uncharacterized | no |
| RRTN | SYN30436 | *Zm00001d013786* | Glutamate formiminotransferase 1 | GO:0005542; GO:0016740 |
| RRTN | SYN30436 | *Zm00001d013787* | Secretory carrier-associated membrane | GO:0015031 |
| RRTN | SYN30436 | *Zm00001d013788* | Secretory carrier-associated membrane | GO:0015031 |
| RRTN | SYN30436 | *Zm00001d013790* | Uncharacterized | no |
| RRTN | SYN30436 | *Zm00001d013792* | Agamous-like MADS-box protein AGL80 | GO:0000981; GO:0046983; GO:0000978; GO:0045944; GO:0006357 |
| RRTN | SYN30436 | *Zm00001d013793* | HD domain-containing metal-dependent phosphohydrolase | GO:0008832; GO:0046872; GO:0006203 |
| RRTN | SYN30436 | *Zm00001d013794* | Uncharacterized | no |
| RRTN | SYN30436 | *Zm00001d013795* | Peroxisomal membrane 22 kDa (Mpv17/PMP22) | no |
| RRTN | SYN30436 | *Zm00001d013796* | Dicer-like 105 | GO:0005524; GO:0004525; GO:0003723; GO:0048856; GO:0035194; GO:0070918 |
| RRTN | SYN30436 | *Zm00001d013797* | Endoribonuclease Dicer homolog 2 | GO:0004525; GO:0003723; GO:0016075; GO:0030422 |
| RRTN | SYN30436 | *Zm00001d013798* | Cycloartenol synthase | GO:0016866; GO:0016104 |
| RRTN | SYN30436 | *Zm00001d013799* | Membrane metalloprotease ARASP chloroplastic | GO:0004222 |
| RRTN | SYN30436 | *Zm00001d013800* | Uncharacterized | no |
| RRTN | SYN30436 | *Zm00001d013801* | Myb domain protein | GO:0003677 |
| RRTN | SYN30436 | *Zm00001d013804* | Hexosyltransferase | GO:0008378; GO:0016757; GO:0008194; GO:0006486 |
| RRTN | SYN30436 | *Zm00001d013806* | Uncharacterized | no |
| RRTN | SYN30436 | *Zm00001d013808* | Protein REDUCED WALL ACETYLATION 2 | GO:0016407; GO:0005975; GO:0009834; GO:1990937; GO:0045492; GO:0010411 |
| RRTN | SYN30436 | *Zm00001d013809* | Protein DETOXIFICATION | GO:0015297; GO:0022857; GO:0042910; GO:1990961 |
| RRTN | SYN30436 | *Zm00001d013810* | Protein DETOXIFICATION | GO:0015297; GO:0022857; GO:0042910; GO:1990961 |
| RRTN | SYN30436 | *Zm00001d013811* | Protein DETOXIFICATION | GO:0015297; GO:0022857; GO:0042910; GO:1990961 |
| RRTN | SYN30436 | *Zm00001d013812* | Polyadenylate-binding protein RBP45C | GO:0003723 |
| RRTN | SYN30436 | *Zm00001d013814* | Pentatricopeptide repeat-containing protein | no |
| RRTN | SYN30436 | *Zm00001d013815* | Condensin-2 complex subunit H2 | GO:0030261 |
| RRTN | SYN30436 | *Zm00001d013816* | tRNA-uridine aminocarboxypropyltransferase | GO:0016432; GO:0008033 |
| RRTN | SYN30436 | *Zm00001d013817* | Protein FATTY ACID EXPORT 5 | GO:0015245; GO:0015908 |
| RRTN | SYN30436 | *Zm00001d013818* | Tryptophan N-monooxygenase 2 | GO:0005506; GO:0020037 |
| RRTN | SYN30436 | *Zm00001d013819* | Galactose mutarotase-like superfamily protein | GO:0030246; GO:0016853; GO:0005975 |
| RRTN | SYN30436 | *Zm00001d013820* | Uncharacterized | no |
| RRTN | SYN30436 | *Zm00001d013821* | Methyltransf_21 domain-containing protein | no |
| RRTN | SYN30436 | *Zm00001d013822* | Putative disease resistance RPP13-like protein 1 | GO:0043531; GO:0005524; GO:0006952 |
| RRTN | SYN30436 | *Zm00001d013823* | L-gulonolactone oxidase | GO:0003885 ; GO:0071949; GO:0016491; GO:0050105; GO:0019853 |
| RRTN | SYN30436 | *Zm00001d013824* | F-box domain containing protein expressed | no |
| RRTN | SYN30436 | *Zm00001d013825* | Uncharacterized | no |
| RRTN | SYN30436 | *Zm00001d013827* | TRF-like 5 | GO:0003677 |
| RRTN | SYN30436 | *Zm00001d013828* | Switch 2 | GO:0005524; GO:0140658 |
| SL | SYN24465 | *Zm00001d001453* | Uncharacterized | no |
| SL | SYN24465 | *Zm00001d038301* | Uncharacterized | no |
| SL | SYN24465 | *Zm00001d038302* | Protein ROS1 | GO:0051539; GO:0003677; GO:0035514; GO:0019104; GO:0046872; GO:0006284 |
| SL | SYN24465 | *Zm00001d038303* | Uncharacterized | no |
| SL | SYN24465 | *Zm00001d038304* | Carbohydrate-binding-like fold | GO:2001070 |
| SL | SYN24465 | *Zm00001d038306* | Cytoplasmic membrane protein | no |
| SL | SYN24465 | *Zm00001d038307* | Glutathione S-transferase family protein | GO:0016740 |
| SL | SYN24465 | *Zm00001d038308* | Uncharacterized | GO:0030276; GO:0072318; GO:0072583 |
| SL | SYN24465 | *Zm00001d038309* | Cyclin-related | no |
| SL | SYN24465 | *Zm00001d038310* | Tubby-like F-box protein | no |
| SL | SYN24465 | *Zm00001d038311* | BZIP transcription factor | GO:0003700; GO:0009738; GO:0045893 |
| SL | SYN24465 | *Zm00001d038312* | Protein YIP | GO:0006888 |
| SL | SYN24465 | *Zm00001d038314* | ATP-dependent DNA helicase | GO:0005524; GO:0016887; GO:0003678; GO:0006310; GO:0006281; GO:0000723 |
| SL | SYN24465 | *Zm00001d038316* | Uncharacterized | no |
| SL | SYN24465 | *Zm00001d038317* | Nicastrin | GO:0016485 |
| SL | SYN24465 | *Zm00001d038318* | Polypyrimidine tract-binding protein homolog 3 | GO:0003723; GO:0006397 |
| SL | SYN24465 | *Zm00001d038319* | Protein LIKE COV 2 | no |
| SL | SYN24465 | *Zm00001d038321* | S-acyltransferase | GO:0019706; GO:0018230; GO:0006612 |
| SL | SYN24465 | *Zm00001d038323* | Uncharacterized | no |
| SL | SYN24465 | *Zm00001d038324* | Uncharacterized | no |
| SL | SYN24465 | *Zm00001d038325* | Uncharacterized | no |
| SL | SYN24465 | *Zm00001d038326* | Serine/threonine-protein kinase SRK2A | GO:0005524; GO:0004674; GO:0035556; GO:0006468 |
| SL | SYN24465 | *Zm00001d038327* | Pi starvation-induced protein | GO:0010951 |
| SL | SYN24465 | *Zm00001d038328* | Alpha/beta-Hydrolases superfamily protein | GO:0047372; GO:0034338; GO:0044255 |
| SL | SYN24465 | *Zm00001d038329* | Transmembrane protein 18 | no |
| SL | SYN24465 | *Zm00001d038330* | DUF1682 family protein | GO:0005509; GO:0032469 |
| SL | SYN24465 | *Zm00001d038331* | Uncharacterized | no |
| SL | SYN24465 | *Zm00001d038332* | Amidophosphoribosyltransferase | GO:0004044; GO:0051536; GO:0046872; GO:0006189; GO:0009113; GO:0006164 |
| SL | SYN24465 | *Zm00001d038333* | Protein NRT1/ PTR FAMILY 5.10 | GO:0022857; GO:0006857 |
| SL | SYN24465 | *Zm00001d038334* | Protein NRT1/ PTR FAMILY 5.10 | GO:0022857 |
| SL | SYN24465 | *Zm00001d038336* | Uncharacterized | no |
| SL | SYN24465 | *Zm00001d038337* | DAR GTPase 3 chloroplastic | GO:0003924; GO:0005525 |
| SL | SYN24465 | *Zm00001d038338* | Transcription repressor MYB6 | GO:0003677 |
| SL | SYN24465 | *Zm00001d038339* | UDP-glucuronate decarboxylase | GO:0070403; GO:0048040 ; GO:0042732; GO:0033320 |
| SL | SYN24465 | *Zm00001d038340* | Phosphoribosylanthranilate transferase | GO:0016740 |
| SL | SYN24465 | *Zm00001d038341* | PAR1 protein | no |
| SL | SYN24465 | *Zm00001d038342* | RING/FYVE/PHD zinc finger superfamily protein | GO:0008270 |
| SL | SYN24465 | *Zm00001d038343* | Phosphatidylinositol:ceramide inositolphosphotransferase 1 | GO:0047493; GO:0045140; GO:0033188; GO:0046513 |
| SL | SYN24465 | *Zm00001d038345* | Lysine-ketoglutarate reductase/saccharopine dehydrogenase bifunctional enzyme | GO:0016758; GO:0006488 |
| SL | SYN24465 | *Zm00001d038346* | Uncharacterized | no |
| SL | SYN24465 | *Zm00001d038347* | TTF-type zinc finger protein with HAT dimerization domain | no |
| SL | SYN24465 | *Zm00001d038348* | Guanylyl cyclase 1 | no |
| RDW | PZE-109058967 | *Zm00001d027142* | Uncharacterized | no |
| RDW | PZE-109058967 | *Zm00001d027143* | Uncharacterized | no |
| RDW | PZE-109058967 | *Zm00001d027144* | Uncharacterized | no |
| RDW | PZE-109058967 | *Zm00001d046571* | Putative HLH DNA-binding domain superfamily protein | GO:0003677; GO:0003700; GO:0046983; GO:0055072 |
| RDW | PZE-109058967 | *Zm00001d046574* | Uncharacterized | no |
| RDW | PZE-109058967 | *Zm00001d046576* | Protein argonaute 13 | GO:0048856 |
| RDW | PZE-109058967 | *Zm00001d046577* | Cation_ATPase_C domain-containing protein | GO:0019829; GO:0005388 |
| RDW | PZE-109058967 | *Zm00001d046579* | Transcription initiation factor TFIID subunit | GO:0003743 |
| RDW | PZE-109058967 | *Zm00001d046580* | Sterile alpha motif (SAM) domain-containing protein | no |
| RDW | PZE-109058967 | *Zm00001d046581* | Isoleucine--tRNA ligase | GO:0002161; GO:0005524; GO:0004822; GO:0000049; GO:0006428 |
| RDW | PZE-109058967 | *Zm00001d046582* | Uncharacterized | no |
| RDW | PZE-109058967 | *Zm00001d046583* | 40S ribosomal protein S14 | GO:0003735; GO:0006412 |
| RDW | PZE-109058967 | *Zm00001d046585* | Uncharacterized | no |
| RDW | PZE-109058967 | *Zm00001d046586* | Transmembrane amino acid transporter family protein | GO:0015171; GO:0003333 |
| RDW | PZE-109058967 | *Zm00001d046587* | WD-40 repeat family protein | GO:0006364 |
| RDW | PZE-109058967 | *Zm00001d046588* | Uncharacterized | no |
| RDW | PZE-109058967 | *Zm00001d046590* | Uncharacterized | no |
| RDW | PZE-109058967 | *Zm00001d046592* | S-acyltransferase | GO:0019706 |
| RDW | PZE-109058967 | *Zm00001d046593* | Purple acid phosphatase | GO:0003993; GO:0046872; GO:0055062; GO:0051174; GO:0006950 |
| RDW | PZE-109058967 | *Zm00001d046594* | Uncharacterized | no |
| RDW | PZE-109058967 | *Zm00001d046595* | Sucrose-phosphate synthase | GO:0046524; GO:0016157; GO:0005986 |
| RDW | PZE-109058967 | *Zm00001d046596* | Bifunctional inhibitor | no |
| RDW | PZE-109058967 | *Zm00001d046597* | Uncharacterized | no |
| RDW | PZE-109058967 | *Zm00001d046598* | Uncharacterized | no |
| RDW | PZE-109058967 | *Zm00001d046599* | Superal1 | GO:0032509; GO:0045324; GO:0015031 |
| RDW | PZE-109058967 | *Zm00001d046600* | Proteasome subunit beta | GO:0004298; GO:0010498; GO:0043161 |
| RDW | PZE-109058967 | *Zm00001d046601* | Phox (PX) domain-containing protein | GO:0035091; GO:0015031 |
| RDW | PZE-109058967 | *Zm00001d046602* | CUE domain containing protein | GO:0043130 |
| RDW | PZE-109058967 | *Zm00001d046603* | Putative cytochrome P450 superfamily protein | GO:0020037; GO:0005506; GO:0004497; GO:0016705 |
| RDW | PZE-109058967 | *Zm00001d046604* | (Z)-3-hexen-1-ol acetyltransferase | GO:0016747 |
| RDW | PZE-109058967 | *Zm00001d046605* | Uncharacterized | no |
| RDW | PZE-109058967 | *Zm00001d046606* | Uncharacterized | no |
| RDW | PZE-109058967 | *Zm00001d046607* | Uncharacterized | no |
| RDW | PZE-109058967 | *Zm00001d046608* | Uncharacterized | no |
| RDW | PZE-109058967 | *Zm00001d046609* | Uncharacterized | no |
| RDW | PZE-109058967 | *Zm00001d046610* | (Z)-3-hexen-1-ol acetyltransferase | GO:0016747 |
| SL | PZE-106099144 | *Zm00001d001447* | Uncharacterized | no |
| SL | PZE-106099144 | *Zm00001d001448* | Uncharacterized | no |
| SL | PZE-106099144 | *Zm00001d001449* | Uncharacterized | no |
| SL | PZE-106099144 | *Zm00001d001450* | Uncharacterized | no |
| SL | PZE-106099144 | *Zm00001d038235* | Uncharacterized | no |
| SL | PZE-106099144 | *Zm00001d038236* | Uncharacterized | no |
| SL | PZE-106099144 | *Zm00001d038237* | Uncharacterized | no |
| SL | PZE-106099144 | *Zm00001d038239* | Uncharacterized | no |
| SL | PZE-106099144 | *Zm00001d038240* | CSC1-like protein | GO:0005227 |
| SL | PZE-106099144 | *Zm00001d038241* | Uncharacterized | no |
| SL | PZE-106099144 | *Zm00001d038242* | Uncharacterized | no |
| SL | PZE-106099144 | *Zm00001d038244* | Auxin-responsive protein | GO:0009734 |
| SL | PZE-106099144 | *Zm00001d038245* | Uncharacterized | no |
| SL | PZE-106099144 | *Zm00001d038246* | Uncharacterized | no |
| SL | PZE-106099144 | *Zm00001d038247* | Uncharacterized | no |
| SL | PZE-106099144 | *Zm00001d038248* | RNA-binding (RRM/RBD/RNP motifs) family protein | no |
| SL | PZE-106099144 | *Zm00001d038250* | DnaJ protein | GO:0044444; GO:0008150; GO:0005737; GO:0005575; GO:0044464; GO:0005623; GO:0005829; GO:0003674; GO:0044424; GO:0009987; GO:0005622; GO:0005488; GO:0005515 |
| SL | PZE-106099144 | *Zm00001d038251* | Adenine nucleotide alpha hydrolase-like superfamily protein | GO:0016787 |
| SL | PZE-106099144 | *Zm00001d038252* | Potassium channel AKT2/3 | GO:0005249 |
| SL | PZE-106099144 | *Zm00001d038253* | DUF674 family protein | no |
| SL | PZE-106099144 | *Zm00001d038255* | Uncharacterized | no |
| SL | PZE-106099144 | *Zm00001d038256* | Uncharacterized | no |
| SL | PZE-106099144 | *Zm00001d038257* | Pentatricopeptide repeat-containing protein | no |
| SL | PZE-106099144 | *Zm00001d038258* | Uncharacterized | GO:0003712; GO:0006355 |
| SL | PZE-106099144 | *Zm00001d038260* | FAM91A1-like protein | no |
| SL | PZE-106099144 | *Zm00001d038262* | Patellin-1 | GO:0008289; GO:0007049; GO:0051301 |
| SL | PZE-106099144 | *Zm00001d038263* | O-fucosyltransferase family protein | GO:0016757; GO:0006004 |
| SL | PZE-106099144 | *Zm00001d038264* | Uncharacterized | no |
| SL | PZE-106099144 | *Zm00001d038266* | Small nuclear RNA activating complex (SNAPc) subunit SNAP43 protein | GO:0043565; GO:0042795; GO:0042796 |
| SL | PZE-106099144 | *Zm00001d038267* | Maternal effect embryo arrest 59 | no |
| SL | PZE-106099144 | *Zm00001d038268* | Putative anion transporter 3 chloroplastic | GO:0005315 |
| SL | PZE-106099144 | *Zm00001d038269* | UPF0503 protein chloroplastic | no |
| SL | PZE-106099144 | *Zm00001d038270* | Duplicated homeodomain-like superfamily protein | GO:0003677 |
| SL | PZE-106099144 | *Zm00001d038271* | Beta-glucosidase | GO:0008422; GO:0102483; GO:0009251 |
| SL | PZE-106099144 | *Zm00001d038272* | Beta-glucosidase | GO:0008422; GO:0102483; GO:0009251 |
| SL | PZE-106099144 | *Zm00001d038273* | Coronatine-insensitive protein 1 | no |
| SL | PZE-106099144 | *Zm00001d038274* | Tetratricopeptide repeat (TPR)-like superfamily protein | no |
| SL | PZE-106099144 | *Zm00001d038275* | Auxin transporter-like protein 1 | GO:0006865 |
| SL | PZE-106099144 | *Zm00001d038276* | Uncharacterized | no |
| SL | PZE-106099144 | *Zm00001d038278* | Uncharacterized | no |
| SL | PZE-106099144 | *Zm00001d038279* | Nuclear ribonuclease Z | no |
| SL | PZE-106099144 | *Zm00001d038280* | U-box domain-containing protein 29 | GO:0061630 |
| SL | PZE-106099144 | *Zm00001d038281* | Dof zinc finger protein DOF1.2 | GO:0003700; GO:0003677 |
| SL | PZE-106099144 | *Zm00001d038282* | Putative LSTK-1-like/NimA-related protein kinase family protein isoform 1 | GO:0005524; GO:0004674; GO:0007017 |
| SL | PZE-106099144 | *Zm00001d038335* | OSJNBb0008G24.13-like protein | GO:0022857 |
| RAD | PZE-103084802/PZE-103084794 | *Zm00001d041755* | ATP-dependent DNA helicase | GO:0005524; GO:0016887; GO:0003678; GO:0006310; GO:0006281; GO:0000723 |
| RAD | PZE-103084802/PZE-103084794 | *Zm00001d041756* | 60S ribosomal protein L6 | GO:0003723; GO:0003735; GO:0002181; GO:0000027 |
| RAD | PZE-103084802/PZE-103084794 | *Zm00001d041758* | eIF-2B GDP-GTP exchange factor subunit alpha | GO:0003743; GO:0050790; GO:0006413 |
| RAD | PZE-103084802/PZE-103084794 | *Zm00001d041759* | Transducin/WD40 repeat-like superfamily protein | no |
| RAD | PZE-103084802/PZE-103084794 | *Zm00001d041760* | 30S ribosomal protein S4, chloroplastic | GO:0019843; GO:0003735; GO:0045903; GO:0006412 |
| RAD | PZE-103084802/PZE-103084794 | *Zm00001d041762* | Putative pentatricopeptide repeat-containing protein | GO:0003723; GO:0009451 |
| RAD | PZE-103084802/PZE-103084794 | *Zm00001d041763* | Glycosyltransferase | GO:0080043; GO:0080044 |
| RAD | PZE-103084802/PZE-103084794 | *Zm00001d041768* | NEP-interacting protein 1 | no |
| RAD | PZE-103084802/PZE-103084794 | *Zm00001d041769* | Carboxypeptidase | GO:0016747; GO:0004185; GO:0019748 |
| RAD | PZE-103084802/PZE-103084794 | *Zm00001d041770* | Uncharacterized | no |
| RAD | PZE-103084802/PZE-103084794 | *Zm00001d041772* | Glutathione transferase | GO:0004364; GO:0009072; GO:0006749 |
| RAD | PZE-103084802/PZE-103084794 | *Zm00001d041773* | Leucine-rich repeat protein kinase family protein | GO:0005524; GO:0004674 |
| RAD | PZE-103084802/PZE-103084794 | *Zm00001d041774* | ARGOS9 | GO:0046622; GO:0009725 |
| RAD | PZE-103084802/PZE-103084794 | *Zm00001d041775* | 3-deoxy-8-phosphooctulonate synthase | GO:0008676; GO:0046364 |
| RAD | PZE-103084802/PZE-103084794 | *Zm00001d041776* | Dhurrinase-like B-glucosidase | GO:0008422; GO:0005975 |
| RAD | PZE-103084802/PZE-103084794 | *Zm00001d041777* | Beta-glucosidase 17 | GO:0008422; GO:0005975 |
| RAD | PZE-103084802 | *Zm00001d041766* | Transcription initiation factor TFIID subunit 8 | GO:0046982; GO:0003743 |
| RAD | PZE-103084794 | *Zm00001d041752* | ARF guanine-nucleotide exchange factor GNL2 | GO:0005085; GO:0009846; GO:0032012 |
| RAD | PZE-103084794 | *Zm00001d041753* | SSXT protein | GO:0008283; GO:0048366; GO:0045944 |
| RAD | PZE-103084794 | *Zm00001d041767* | Alfin-like transcription factor | GO:0042393; GO:0046872; GO:0006325; GO:0006355 |
| RDW | bt2.5/bt2.8 | *Zm00001d026826* | Uncharacterized | no |
| RDW | bt2.5/bt2.8 | *Zm00001d026827* | Uncharacterized | no |
| RDW | bt2.5/bt2.8 | *Zm00001d050102* | Uncharacterized | no |
| RDW | bt2.5/bt2.8 | *Zm00001d050103* | Protein trichome birefringence-like 16 | GO:0016740 |
| RDW | bt2.5/bt2.8 | *Zm00001d050104* | Uncharacterized | no |
| RDW | bt2.5/bt2.8 | *Zm00001d050106* | Ferredoxin-related | no |
| RDW | bt2.5/bt2.8 | *Zm00001d050107* | 12-oxo-phytodienoic acid reductase | GO:0016629; GO:0010181; GO:0016491; GO:0006633; GO:0009695; GO:0031408 |
| RDW | bt2.5/bt2.8 | *Zm00001d050111* | Zinc finger protein | GO:0003700; GO:0009739; GO:0010090 |
| RDW | bt2.5/bt2.8 | *Zm00001d050112* | Putative DUF1296 domain containing family protein | no |
