# Supplementary material for: GWAS and RNA-seq analysis uncover candidate genes associated with alkaline stress tolerance in maize (Zea mays L.) seedlings
Source: Front Plant Sci. 2022 Jul 18;13:963874. doi: 10.3389/fpls.2022.963874 (PMC9340071; doi:10.3389/fpls.2022.963874)
Supplement: Supplementary file 1 [file Data_Sheet_1.zip › Table s6.docx]

**Supplementary file 7：**

**Table S6:** Protein accession number for constructing phylogenetic tree with Zm00001d038250 protein.

| Name | Species | NCBI accession number |
| --- | --- | --- |
| Zm00001d038250 | *Zea mays* | NP_001141369.1 |
| ZmPWZ18936.1 | *Zea mays* | PWZ18936.1 |
| ZmACG28517.1 | *Zea mays* | ACG28517.1 |
| MlCAD6333748.1 | *Miscanthus lutarioriparius* | CAD6333748.1 |
| SbXP_002441136.1 | *Sorghum bicolor* | XP_002441136.1 |
| SbKAG0518188.1 | *Sorghum bicolor* | KAG0518188.1 |
| DoOEL22173.1 | *Dichanthelium oligosanthes* | OEL22173.1 |
| SiXP_004961994.1 | *Setaria italica* | XP_004961994.1 |
| PhXP_025805351.1 | *Panicum hallii* | XP_025805351.1 |
| PhPUZ65880.1 | *Panicum hallii var. hallii* | PUZ65880.1 |
| PvXP_039838234.1 | *Panicum virgatum* | XP_039838234.1 |
| PvXP_039800887.1 | *Panicum virgatum* | XP_039800887.1 |
| PmRLM98454.1 | *Panicum miliaceum* | RLM98454.1 |
| EcGJM88660.1 | *Eleusine coracana subsp. coracana* | GJM88660.1 |
| EcGJN14477.1 | *Eleusine coracana subsp. coracana* | GJN14477.1 |
| EcTVU19471.1 | *Eragrostis curvula* | TVU19471.1 |
| ZpKAG8085657.1 | *Zizania palustris* | KAG8085657.1 |
| TdXP_037417173.1 | *Triticum dicoccoides* | XP_037417173.1 |
| TdXP_037471169.1 | *Triticum dicoccoides* | XP_037471169.1 |
| OmKAF0926957.1 | *Oryza meyeriana var. granulata* | KAF0926957.1 |
| LrXP_047045644.1 | *Lolium rigidum* | XP_047045644.1 |
| ObXP_040380523.1 | *Oryza brachyantha* | XP_040380523.1 |
| LrXP_047045685.1 | *Lolium rigidum* | XP_047045685.1 |
| DeKAF8779432.1 | *Digitaria exilis* | KAF8779432.1 |
| ZpKAG8044137.1 | *Zizania palustris* | KAG8044137.1 |
| ObXP_015690541.2 | *Oryza brachyantha* | XP_015690541.2 |
| TaXP_044450505.1 | *Triticum aestivum* | XP_044450505.1 |
| ZpKAG8068587.1 | *Zizania palustris* | KAG8068587.1 |
| OsXP_015622600.1 | *Oryza sativa Japonica Group* | XP_015622600.1 |
| OsBAB86234.1 | *Oryza sativa Japonica Group* | BAB86234.1 |
