# Supplementary material for: GWAS and RNA-seq analysis uncover candidate genes associated with alkaline stress tolerance in maize (Zea mays L.) seedlings
Source: Front Plant Sci. 2022 Jul 18;13:963874. doi: 10.3389/fpls.2022.963874 (PMC9340071; doi:10.3389/fpls.2022.963874)
Supplement: Supplementary file 1 [file Data_Sheet_1.zip › Table s7.docx]

**Supplementary file 8：**

**Table S7:** Protein accession number for constructing phylogenetic tree with Zm00001d001960 protein.

| Name | species | NCBI accession number |
| --- | --- | --- |
| Zm00001d001960 | *Zea mays* | NP_001130275.1 |
| ZmPWZ37934.1 | *Zea mays* | PWZ37934.1 |
| ZmACG38441.1 | *Zea mays* | ACG38441.1 |
| ZmAAA91227.1 | *Zea mays* | AAA91227.1 |
| SbXP_002448703.2 | *Sorghum bicolor* | XP_002448703.2 |
| SbEES13031.1 | *Sorghum bicolor* | EES13031.1 |
| SbKXG27349.1 | *Sorghum bicolor* | KXG27349.1 |
| MlCAD6261881.1 | *Miscanthus lutarioriparius* | CAD6261881.1 |
| SbKAG0527950.1 | *Sorghum bicolor* | KAG0527950.1 |
| SbKAG0527951.1 | *Sorghum bicolor* | KAG0527951.1 |
| MlCAD6259186.1 | *Miscanthus lutarioriparius* | CAD6259186.1 |
| SbADB66755.1 | *Sorghum bicolor* | ADB66755.1 |
| DeCAB3488565.1 | *Digitaria exilis* | CAB3488565.1 |
| PmRLM65217.1 | *Panicum miliaceum* | RLM65217.1 |
| EcGJN26142.1 | *Eleusine coracana subsp. coracana* | GJN26142.1 |
| SiXP_004960184.1 | *Setaria italica* | XP_004960184.1 |
| SvTKW24058.1 | *Setaria viridis* | TKW24058.1 |
| DeCAB3504162.1 | *Digitaria exilis* | CAB3504162.1 |
| PhXP_025825493.1 | *Panicum hallii* | XP_025825493.1 |
| PhPUZ49342.1 | *Panicum hallii var. hallii* | PUZ49342.1 |
| SvXP_034584802.1 | *Setaria viridis* | XP_034584802.1 |
| PmRLM73296.1 | *Panicum miliaceum* | RLM73296.1 |
| MlCAD6261882.1 | *Miscanthus lutarioriparius* | CAD6261882.1 |
| PvXP_039821892.1 | *Panicum virgatum* | XP_039821892.1 |
| DoOEL16075.1 | *Dichanthelium oligosanthes* | OEL16075.1 |
| IhASU91347.1 | *Indosasa hispida* | ASU91347.1 |
| LrXP_047069543.1 | *Lolium rigidum* | XP_047069543.1 |
| PvXP_039774399.1 | *Panicum virgatum* | XP_039774399.1 |
| TaXP_044320716.1 | *Triticum aestivum* | XP_044320716.1 |
| TaSPT20280.1 | *Triticum aestivum* | SPT20280.1 |
